# New Thiosemicarbazide Derivatives with Multidirectional Biological Action

**DOI:** 10.3390/molecules29071529

**Published:** 2024-03-29

**Authors:** Patryk Lasek, Urszula Kosikowska, Przemysław Kołodziej, Grażyna Kubiak-Tomaszewska, Natalia Krzyżanowska, Tomasz Szostek, Marta Struga, Marcin Feldo, Anna Bogucka-Kocka, Monika Wujec

**Affiliations:** 1 Doctoral School, Medical University of Lublin, Chodzki 7, 20-093 Lublin, Poland; lasekpatryk2@gmail.com; 2 Department of Pharmaceutical Microbiology, Faculty of Pharmacy, Medical University, 20-093 Lublin, Poland; urszula.kosikowska@umlub.pl; 3 Department of Biology and Genetics, Faculty of Pharmacy, Medical University of Lublin, 4A Chodzki Street, 20-093 Lublin, Poland; przemyslawkolodziej@umlub.pl (P.K.); natalia.krzyzanowska97@gmail.com (N.K.); anna.bogucka-kocka@umlub.pl (A.B.-K.); 4 Department of Biochemistry, Medical University of Warsaw, 02-097 Warszawa, Poland; grazyna.kubiak-tomaszewska@wum.edu.pl (G.K.-T.); tomasz.szostek@wum.edu.pl (T.S.); marta.struga@wum.edu.pl (M.S.); 5 Department of Vascular Surgery, Medical University of Lublin, Staszica 11 St., 20-081 Lublin, Poland; marcin.feldo@umlub.pl; 6 Department of Organic Chemistry, Faculty of Pharmacy, Medical University of Lublin, 4A Chodzki Street, 20-093 Lublin, Poland

**Keywords:** synthesis, thiosemicarbazides, antibacterial activity, nematocidal activity, cytotoxic effect, antioxidant activity

## Abstract

Over the years, several new medicinal substances have been introduced for the treatment of diseases caused by bacteria and parasites. Unfortunately, due to the production of numerous defense mechanisms by microorganisms and parasites, they still pose a serious threat to humanity around the world. Therefore, laboratories all over the world are still working on finding new, effective methods of pharmacotherapy. This research work aimed to synthesize new compounds derived from 3-trifluoromethylbenzoic acid hydrazide and to determine their biological activity. The first stage of the research was to obtain seven new compounds, including six linear compounds and one derivative of 1,2,4-triazole. The PASS software was used to estimate the potential probabilities of biological activity of the newly obtained derivatives. Next, studies were carried out to determine the nematocidal potential of the compounds with the use of nematodes of the genus *Rhabditis* sp. and antibacterial activity using the ACCT standard strains. To determine the lack of cytotoxicity, tests were performed on two cell lines. Additionally, an antioxidant activity test was performed due to the importance of scavenging free radicals in infections with pathogenic microorganisms. The conducted research proved the anthelmintic and antibacterial potential of the newly obtained compounds. The most effective were two compounds with a 3-chlorophenyl substituent, both linear and cyclic derivatives. They demonstrated higher efficacy than the drugs used in treatment.

## 1. Introduction

Parasitic diseases continue to affect millions of people worldwide; they still pose a great challenge to public health and are a threat to livestock resources. A particular group of parasites are helminths, which include nematodes (round worms), trematodes (flat worms), and cestodes (tapeworms). The World Health Organization (WHO) classifies diseases caused by helminths, such as schistosomiasis, lymphatic filariasis, dracunculiasis, and soil-transmitted helminth infections, among the neglected tropical diseases, which affect multiple populations and cause disability and suffering. Those diseases could be controlled; however, scarce funding is devoted to them. Developing countries in which these diseases are endemic are often unable to afford research on new potential anthelmintic agents [1]. According to the Centers for Disease Control and Prevention (CDC), a large part of the world’s population is infected with soil-transmitted helminths (approximately 807–1121 million with *Ascaris lumbricoides*, 604–795 million with *Trichuris trichuria*, and 576–740 million with *Necator americanus* or *Ancylostoma duodenale*) [2].

Furthermore, there are only a few classes of anthelmintic drugs, which have multiple disadvantages, such as side effects, and the majority of them were introduced many years ago. An additional problem is widespread resistance to the medicines that are currently used, especially for the agricultural sections of both industrialized and developing countries’ economies [3]. The development of resistance, which has been reported in all classes of veterinary anthelmintics, is a result of the intensive application of antiparasitic drugs in light of the general lack of antiparasitic vaccines [4,5]. The mechanisms underlying resistance include upregulation of cellular efflux processes, enhanced drug metabolism, reduced receptor expression, or changes in drug receptor sites that reduce drug binding or receptor functions; for instance, mutations in the beta-tubulin gene may cause nematodes to develop resistance to benzimidazoles [6,7]. Given the above, there is a great necessity not only to detect, monitor, and investigate resistance mechanisms but also to search for new compounds that demonstrate antiparasitic activity. A great proportion of drugs have been introduced as an effect of known compound modification. The purpose of this work was to analyze the nematocidal properties of newly obtained thiosemicarbazide derivatives containing a trifluoromethyl group. The in vitro efficacy of these compounds was evaluated against free-living nematodes—*Rhabditis* sp.—as model organisms, with albendazole as a reference drug. Moreover, the antibacterial in vitro activity of newly synthesized compounds was investigated towards both Gram-positive and Gram-negative bacteria.

In recent years, etiological agents of infective diseases and new resistance mechanisms developed by bacteria have greatly reduced the number of available therapeutic options and antimicrobial agents effective against bacteria. Frequently, microorganisms mainly isolated from hospitalized patients but also in community-acquired infections show resistance to more than one group of antimicrobials. These microorganisms, known as multi-drug-resistant (MDR) bacteria have become a major problem for global health, and for this reason, the search for new compounds with antimicrobial activity is now a priority for researchers. According to the WHO, *Staphylococcus aureus* is one of the priority opportunistic bacteria with known pathogenicity for research on new antimicrobials. This microorganism can induce toxicoinfections and diseases of the soft tissues and skin, as well as the respiratory, urinary, or digestive system. In turn, *Staphylococcus epidermidis*, which colonizes the human body as a microbiota element in parts of the mucous membranes and skin, can be the major etiological factor of nosocomial infections [8,9].

Drugs used in both bacterial infections and parasitic diseases are often toxic. When looking for new structures, you should pay attention to the safety of their use. That is why cytotoxicity tests are performed. Reactive oxygen species are produced in many chemical reactions related to cellular metabolism but also as the body’s response to bacterial infections. ROS can cause oxidation of proteins and lipids, DNA damage, destruction of extracellular matrix components such as collagen, hyaluronans, and proteoglycans, as well as the induction of metalloproteinases [10]. For this reason, antioxidant activity is desirable for compounds with antimicrobial activity.

Thiosemicarbazides are compounds with a broad spectrum of biological activity. Their antimicrobial properties deserve special attention [11,12,13,14,15,16,17,18,19,20,21,22,23,24,25]. Derivatives with antiviral, antimalarial, and anticancer activity are also known [26,27,28,29,30]. Thiosemicarbazides are often an initial compound for the synthesis of new active substances that include antibacterial and antifungal properties [31,32,33,34,35,36].

The introduction of fluorine or trifluoromethyl groups into organic molecules, particularly in pharmaceuticals, has been a well-documented strategy in medicinal chemistry [37]. These modifications can lead to significant improvements in the pharmacokinetic and physicochemical properties of the molecules. Some of the benefits include enhanced metabolic stability by resisting enzymatic degradation and improving the lipophilicity of the molecule, which can enhance its ability to cross cellular membranes. Additionally, fluorine substitution can influence the binding interactions of a molecule with its target protein, potentially leading to increased potency or selectivity.

Considering the above, in the presented work, we proposed the synthesis of new thiosemicarbazide derivatives with a trifluoromethylphenyl substituent as promising antiparasitic, antibacterial, and antioxidant agents.

## 2. Results and Discussion

### 2.1. Chemistry

Based on the tests carried out in our research group, it can be stated with certainty that thiosemicarbazide derivatives are characterized by higher antimicrobial activity than their cyclic analog—1,2,4-triazole derivatives. Initially, we assumed that the aim of the work would be the synthesis of thiosemicarbazide derivatives. Based on the obtained results of biological tests, we also decided to synthesize a cyclic analog of the most active thiosemicarbazide derivative to determine the impact of closing the linear system in a 1,2,4-triazole ring on the biological activity.

**Scheme 1:**
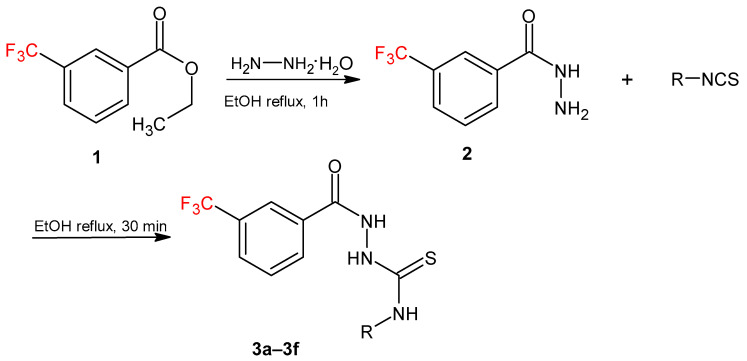
Synthetic route for the compounds **1**–**3**.

To synthesize new derivatives, ethyl 3-trifluoromethylbenzoate (**1**) was used as a substrate. In the reaction with 100% hydrazine hydrate in anhydrous ethanol, 3-trifluoromethylbenzoic acid hydrazide (**2**) was obtained. Then, by reacting with selected isothiocyanates, new thiosemicarbazide derivatives were formed. The reactions were carried out by a known procedure, heating the substrates for 30 min in an ethanol medium [38,39,40]. Substituents for synthesis were selected in such a way that based on the results of the biological studies, it was possible to determine the effect of the type of substituent on the anthelmintic and antimicrobial activity. The new 1-(3-trifluoromethylbenzoyl)thiosemicarbazides **3a**–**3f** were prepared according the Scheme 1.
**Compound Number****R****Compound Number****R****3a**
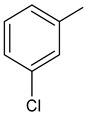
**3d**
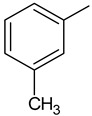
**3b**
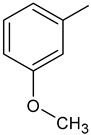
**3e**
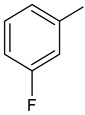
**3c**
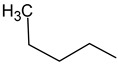
**3f**
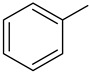


**Scheme 2:**
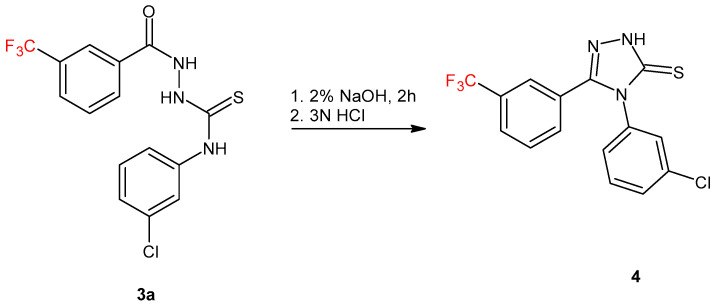
Synthetic route for compound **4**.

The synthesis of the 1,2,4-triazole-3-thione derivative (**4**) consisted of dissolving 4-(3-chlorophenyl)-1-(3-trifluoromethylbenzoyl)thiosemicarbazide in 2% aqueous NaOH solution and heating under reflux for 2 h (Scheme 2).

As a result of the reactions conducted, we obtained six undescribed derivatives of 3-trifluoromethylbenzoic acid thiosemicarbazide (**3a**–**3f**) and one cyclic compound, a 1,2,4-triazole-3-thione derivative (**4**).

The yields of the conducted reactions were in the range of 58–69%. The structures of all compounds were determined by IR and NMR spectra. In the ^1^H NMR spectra of compounds **3a**–**3f**, signals characteristic of aromatic ring protons in the range of 6.80–8.43 ppm were observed. Signals characteristic for protons located on nitrogen atoms in thiosemicarbazide derivatives were observed as two or three singlets in the range of 8.25–10.93 ppm. In compound **4**, a signal for an NH group as a singlet at δ = 14.30 ppm was observed. The ^13^C NMR spectra confirm the structures of the obtained compounds. Measurement of ^19^F NMR spectra was performed for two compounds: one linear (**3a**) and one cyclic (**4**). The signals of three fluorine atoms were observed for compounds **3a** and **4** at δ = −61.11 and −61.66 ppm, respectively.

### 2.2. Predictions of the Biological Activity

Predictions of the potential biological activity of the newly obtained thiosemicarbazide derivatives were carried out using the PASS (Prediction of Activity Spectra for Substances) software [41]. 

Based on the obtained results, it was found that among all the activities, those presented in Table 1 seem to be the most probable.

Based on the obtained results, a decision was made to test the newly synthesized compounds confirming their antiparasitic and antibacterial activity, while confirmation of the antituberculosis activity was left to be carried out in the future due to the limited availability of such studies.

### 2.3. Anthelmintic Activity

**Figure 1:**
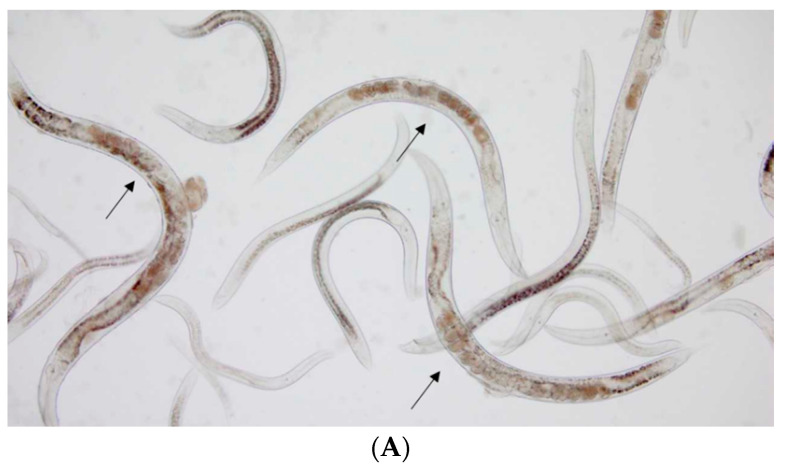

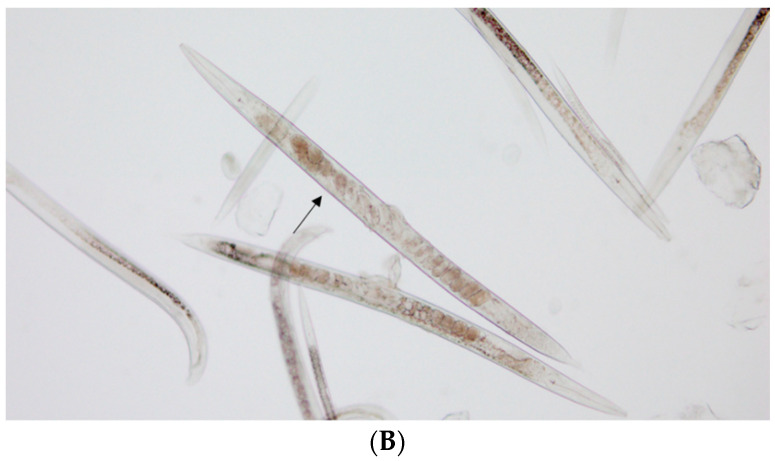
Example of a culture of nematodes of the genus *Rhabditis* sp. (**A**) control (arrow marked alive); (**B**) after the action of the compound **4** (arrow marked dead).

Two of the tested compounds showed anthelmintic activity: 4-(3-chlorophenyl)-5-(3-trifluoromethylphenyl)-2,4-dihydro-3*H*-1,2,4-triazole-3-thione (**4**) and 4-(3-chlorophenyl)-1-(3-trifluoromethylbenzoyl)thiosemicarbazide (**3a**). Both had a 3-chlorophenyl substituent. The anthelmintic activity of the 1,2,4-triazole derivative (**4**) was better than the thiosemicarbazide derivative (**3a**). The LC_50_ value for compound **4** was 0.37 mg/mL, whereas for compound **3a** it was 14.77 mg/mL. Figure 1 shows the nematode culture *Rhabditis* sp. alive, moving (control—A), and dead after the administration of compound **4** (B). The other compounds did not show anthelmintic activity in the concentrations tested. The two active compounds showed anthelmintic activity better than albendazole. The LC_50_ value for this drug was 19.24 mg/mL [42].

In our previous work [43], the most active compounds also had a chlorophenyl substituent, so it seems that it can be said to be a structural element promoting antiparasitic activity. Further, by comparing the antibacterial and antiparasitic activity of new compounds, it can be concluded that in the case of bacteria, the type of substituent has less influence than the structure of the basic skeleton. The linear structure is preferred. However, when analyzing the results of antiparasitic tests, it can be noticed that the type of substituent is of decisive importance, and in this case, the cyclic compounds are characterized by higher activity. Confirmation of these theses will be the subject of our further research.

### 2.4. Antimicrobial Activity

We described the results of experiments that aimed to evaluate the in vitro antibacterial activities of newly synthesized thiosemicarbazides and 1,2,4-triazole derivative. The broth microdilution method to examine the compounds’ ability to inhibit bacterial growth in vitro toward bacterial species from the ATCC (American Type Culture Collection) was used. We used five staphylococci strains including both *Staphylococcus aureus* (four strains) and *Staphylococcus epidermidis* (one strain) species. Staphylococci infections and increasing drug resistance have caused substantial public health problems. Represented by *S. aureus*, ATCC 43300 strain methicillin-resistant bacteria, known as MRSA, are of particular concern because of their ability to spread rapidly and extensively, along with their multi-drug resistance to various antimicrobials, especially β-lactam and aminoglycoside antibiotics [44,45]. MRSA-positive diseases are often connected with chronic or recurrent infections, including respiratory infections, and endocardial inflammation [46].

The minimum inhibitory concentration (MIC) values in µg/mL of the tested compounds against selected Gram-positive bacteria are reported in Table 2.

Newly synthesized compounds were examined for their antimicrobial activity with promising results against selected Gram-positive bacteria. According to our screening results, the highest antibacterial activity against all Gram-positive bacterial strains was demonstrated by thiosemicarbazide **3a**, with an MIC value of 1.95 µg/mL against all *Staphylococcus* spp. strains except methicillin-resistant *S. aureus* (MRSA) ATCC 43300 strain with an MIC value of 3.9 µg/mL. Moreover, the MBC value was 1000 µg/mL towards all tested *S. aureus* reference strains. The lowest MBC value (15.63 µg/mL) was detected against *S. epidermidis* ATCC 12228, *M. luteus* ATCC 10240, and *B. cereus* ATCC 10876 (Table 2). The activity of compound **3a** against staphylococci was bacteriostatic with an MBC/MIC ratio >4. This compound has a chlorine atom in position 3 in the phenyl ring.

The slightly less in vitro antibacterial activity against Gram-positive bacteria was demonstrated by thiosemicarbazide **3e** with MIC values in the range of 7.8–31.25 µg/mL and MBC values > 1000 µg/mL. The MIC range for the methicillin-resistant *S. aureus* ATCC 43300 MRSA strain and all methicillin-sensitive (MSSA) *S. aureus* reference strains was 15.63–31.25 µg/mL. The thiosemicarbazide **3e** was most active toward the *B. cereus* ATCC 10876 strain with an MIC value of 7.81 µg/mL. This compound has a fluorine atom in position 3 in the phenyl ring.

The weakest activity against Gram-positive bacteria was shown by thiosemicarbazide compounds **3b**–**3d** and **3f** with MIC ranges from 500 to ≥1000 µg/mL toward seven strains of Gram-positive bacteria (Table 2). Low MICs for these compounds were observed in only one strain of staphylococci—*S. aureus* ATCC 25923. Their MIC values against this strain of *S. aureus* were sequentially as follows: 62.5 µg/mL, 250 µg/mL, 1000 µg/mL, and 62.5 µg/mL. The transformation of the linear system of thiosemicarbazide (**3a**) into a 1,2,4-triazole ring (**4**) in this research results in a drastic decrease in activity in relation to all tested strains.

The antibacterial effect examination showed that all tested compounds were active against *Gram-positive bacteria* and no activity was observed against *Gram-negative strains*. Our data indicate that all tested compounds were ineffective in tested concentrations against the reference strains of Gram-negative bacteria, *Klebsiella pneumoniae* ATCC 13883, *Escherichia coli* ATCC 25922, and *Salmonella typhimurium* ATCC 14028, with MIC values > 1000 µg/mL.

Based on the obtained results, it can be hypothesized that in the studied group of 3-trifluoromethylphenylthiosemicarbazide derivatives, the presence of a halogen in the aromatic ring is a prerequisite for obtaining antibacterial activity. However, comparing the activity of the thiosemicarbazides derived from fluorobenzoic acids obtained earlier, it can be concluded that they were more active than those with a trifluoromethylphenyl substituent. In the future, this should be confirmed by performing tests for compounds with bromine and iodine atoms.

Despite the fact that the obtained activities are at least four times lower than for the reference antibiotic, cefuroxime, they are very good due to the fact that bacteria produce mechanisms of resistance to known drugs and known chemical structures. Thiosemicarbazide derivatives have a structure not yet recognized by microorganisms, which makes them attractive structures for further research. In the future, we will try to determine the impact of the obtained compounds on multidrug-resistant strains.

### 2.5. Anticancer Activity

To establish the antiproliferative activity of newly obtained thiosemicarbazide derivatives, they were tested with an MTT assay using the human colon cancer cell line (SW 620) compared to a normal cell line (Chinese hamster lung cells V79). The cytotoxic activity of the studied compounds is presented in Table 3. IC_50_ values for most of the studied compounds were very high (>100 µM). For compound **3a**, we observed a lower value (IC_50_ = 69.02 ± 12.63 µM for SW620). However, in contrast to the reference compound Doxorubicin (IC_50_ = 1.83 ± 0.10 µM for SW620 and IC_50_ = 0.46 ± 0.03 µM for V79), this result should be considered low as well. Thus, thiosemicarbazide derivatives (**3a**–**3f**) and the tested triazole derivative (**4**) have not caused cytotoxic effects on studied cell lines.

### 2.6. Antioxidant Activity

Oxygen is an element necessary for the life of all aerobic organisms, including humans. Despite its key importance, especially in relation to energy processes and reactions accompanying inflammatory processes, it may pose a threat to cell metabolism. It is known that during the energy processes, including, among others, electron transport chains in mitochondria and microsomes, as well as the activities of enzymes such as superoxide dismutase, flavoprotein oxidases, or xanthine oxidase, oxygen radicals (ROS) may be generated. These include superoxide anions (the main representative of free radicals), hydrogen peroxide (a precursor of free radicals), and hydroxyl radicals. These compounds, which have a high oxidizing potential, are necessary for the proper conduct of many metabolic processes [47,48], but their excessive production may lead to damage to the structure of many macromolecules, such as proteins, nucleic acids, and unsaturated fatty acids [49,50,51].

Naturally occurring non-enzymatic and enzymatic antioxidant systems forming the antioxidant defence system (ADS) protect cells against the effects of ROS by reducing them to water or molecular oxygen and reducing their production [52,53].

Numerous studies have shown the relationship between overproduction of ROS and cancer, as well as cardiovascular, neurodegenerative, and inflammatory diseases [54,55,56,57].

Unsaturated fatty acids are the compounds most susceptible to the effects of ROS, which is particularly dangerous due to their common presence in all biological membranes. Their oxidation products, malondialdehyde (MDA) and trans-4-hydroxynonenal (4HNE), also affect cell metabolism by forming strong bonds with nucleophilic groups in proteins, nucleic acids, and membrane lipids. Both MDA and 4HNE are used as markers in the assessment of cellular oxidative stress [58,59].

The antioxidant potential of the newly synthesized derivatives was assessed based on the analysis of the malondialdehyde (MDA) concentration formed as a product of lipid oxidation stimulated by iron (II) ions [60]. The results are presented in Table 4.

Our research shows that all tested compounds exhibit antioxidant activities and reduced the level of MDA as compared to that in the control sample by 25.1% to 52.8%.

Inactivation of free oxygen radicals occurs as a result of the transfer of a hydrogen atom to this type of compound, the transfer of one electron, or in the process of electron transfer accompanied by the subsequent loss of a proton [61,62].

The presence of sulfur in the structure of thiourea in the tested compounds indicates the possibility of their antioxidant effect through the mechanism of chelation of Fe^2+^ ions, which are the most powerful pro-oxidants [63]. It is known that the introduction of an -OH group to the phenyl ring increases antioxidant activity due to the possibility of creating an aryloxy radical. Also, the introduction of a methoxy group to the phenolic ring significantly increases the antioxidant potential of phenolic acids, as well as stilbenes, flavonoids, and hydroxytyrosol, although it is much lower than in the case of the hydroxyl group [61,64]. The activity enhancement effect is related to the electron-donating properties of the methoxy moiety [65,66]. A similar effect is demonstrated by compound **3d** containing a methoxy group on the phenyl ring compared to compound **3f** containing only the phenyl ring (50.1% antioxidant activity vs. 46.3%). Rimal et al. showed that substituting chalcones with halogens increased their antioxidant activity both in the mechanism of iron ion chelation and free radical scavenging activity [67]. In the case of the tested compounds **3a** and **3e** containing a phenyl ring substituted with chlorine and fluorine, respectively, a slight increase in antioxidant activity was also found compared to compound **3f** containing only the phenyl ring (48.3% and 52.8% of antioxidant activity vs. 46.3%). Cyclization of the thiosemicarbazide (compound **4**) significantly reduces the antioxidant activity compared to compound **3a** (25.1% vs. 48.3%).

Studies on the modification of phenolic compounds in order to obtain higher antioxidant capacity have shown that the introduction of a tert-butyl group into the phenyl structure increases their antioxidant potential. In turn, the introduction of linear substituents, especially long-chain alkanes, does not provide such effects [68]. The introduction of a butyl substituent into the phenyl ring (compound **3c**) significantly reduced the antioxidant activity compared to compound **3f** containing a phenyl ring (34.3% vs. 46.3%).

### 2.7. Lipinski’s Rule of Drug-Likeness

The compounds were filtered with Lipinski’s criteria to check for drug-like characteristics.

Table 5 shows the calculated parameters for thiosemicarbazide derivatives, including the molecular weight, the number of hydrogen bond donors and acceptors, log p (octanol-water partition coefficient), the number of rotational bonds, and the topological polar surface area of atoms in the molecule. Lipinski’s rule is observed by all tested compounds according to the results, indicating that these compounds have the potential to act as active drugs when administered orally.

## 3. Materials and Methods

### 3.1. Chemistry

All the substances used in this work were purchased from Sigma-Aldrich (Munich, Germany) and were used without further purification. On a Fisher-Johns melting point apparatus (Stuart SMP50, Schwerte, Germany), melting points were recorded. Using an elemental analyzer by Perkin-Elmer 2400 CHN (Waltham, MA, USA), elemental analyses of C, H, and N were performed. Using DMSO-*d*_6_ solvent, the ^1^H, ^13^C, and ^19^F NMR spectra were analyzed by using the Bruker Avance 600 (Bruker BioSpin GmbH, Rheinstetten, Germany). In the Supplementary Materials, ^1^H NMR spectra (Figures S1–S7), ^13^C NMR spectra (Figures S8–S14), and ^19^F NMR (Figures S15 and S16) are presented. The progress of the reactions was monitored by TLC (aluminum sheet 60 F254 plates (Merck Co., Kenilworth, NJ, USA). We used the solvent system CHCl_3_/EtOH (10:1, *v*/*v*).

#### 3.1.1. The Procedure for the Synthesis of Hydrazide of 3-Trifluoromethylbenzoic Acid (**2**)

A total of 0.01 mole of ethyl 3-trifluoromethylbenzoate was dissolved in 5 mL anhydrous ethanol, and then 0.02 mole of 100% hydrazine hydrate was added. The solution was refluxed for 1 h. After cooling, the precipitate was filtered off and dried. The yield of the reaction was 99%. Using 80% hydrazine hydrate resulted in obtaining hydrazide with a yield of 80%.

#### 3.1.2. The Procedure for the Synthesis of 1-(3-Trifluoromethylbenzoyl)-4-substituted Thiosemicarbazide (**3a**–**3f**)

A total of 0.001 mol of 3-trifluoromethylbenzoic acid hydrazide (**2**) was dissolved hot in 5 mL of anhydrous ethanol. Then, 0.001 mol of isothiocyanate was added and the mixture was refluxed for 1/2 h. After this time, the contents of the flasks were cooled to room temperature, the precipitates were filtered, washed with water and diethyl ether, and allowed to air dry. The resulting thiosemicarbazide derivatives were purified by crystallization from 96% ethanol.

#### 3.1.3. The Procedure for the Synthesis of 4-(3-Chlorophenyl)-5-(3-trifluoromethylphenyl)-2,4-dihydro-3H-1,2,4-triazole-3-thione (**4**)

A total of 0.1 g of 4-(3-chlorophenyl)-1-(3-trifluoromethylbenzoyl)thiosemicarbazide (**3a**) was dissolved in 5 mL of a 2% aqueous NaOH solution. The solution was refluxed for 2 h. After cooling to room temperature, the solution was treated 3N hydrochloric acid until slightly acidic to litmus paper. The resulting precipitate was filtered off and, after drying, crystallized from 96% ethanol.

##### 4-(3-Chlorophenyl)-1-(3-trifluoromethylbenzoyl)thiosemicarbazide (**3a**)

Yield 65%, m.p. 185.4 °C, ^1^H NMR (DMSO-*d*_6_) δ (ppm): 7.22 (d, 1H, ArH, *J* = 7.9 Hz), 7.36 (t, 1H, ArH, *J* = 8.0 Hz), 7.44 (d, 1H, ArH, *J* = 8.3 Hz), 7.59 (bs, 1H, ArH), 7.78 (t, 1H, ArH, *J* = 8.0 Hz), 7.98 (d, 1H, ArH, *J* = 7.8 Hz), 8.23 (d, 1H, ArH, *J* = 7.9 Hz), 8.29 (s, 1H, ArH), 9.92–9.97 (m, 2H, 2NH), 10.86 (s, 1H, NH). ^13^C NMR (DMSO-*d*_6_) δ (ppm): 123.52, 123.79, 125.03, 125.33, 127.14, 128.93, 128.96, 129.59, 130.17, 132.45, 133.86, 141.15, 141.23, 165.15; 180.78. ^19^F NMR (DMSO-*d*_6_) δ (ppm): −61.11. Elemental analysis for C_15_H_11_ClF_3_N_3_OS. Calculated: C 48.20; H 2.97; N 11.24. Found: C 48.30; H 2.90; N 11.40.

##### 1-(3-Trifluoromethylbenzoyl)-4-(3-methoxyphenyl)thiosemicarbazide (**3b**)

Yield 62%, m.p. 181.3 °C, ^1^H NMR (DMSO-*d*_6_) δ (ppm): 3.80 (s, 3H, CH_3_), 6.80–6.82 (m, 1H, ArH), 7.07–7.08 (m, 1H, ArH), 7.16 (bs, 1H, ArH), 7.30 (t, 1H, ArH *J* = 8.1 Hz) 7.84 (t, 1H, ArH *J* = 7.8 Hz), 8.04 (d, 1H, ArH *J* = 9.1 Hz), 8.29 (d, 1H, ArH *J* = 7.8 Hz), 8.35 (s, 1H, ArH), 9.85 (s, 2H, 2NH), 10.87 (s, 1H, NH). ^13^C NMR (DMSO-*d*_6_) δ (ppm): 55.58, 111.10, 112.24, 118.72, 121.73, 123.54, 125.03, 128.88, 129.34, 130.14, 132.46, 133.99, 140.74, 159.46, 165.17, 181.45. Elemental analysis for C_16_H_14_F_3_N_3_O_2_S. Calculated: C 52.03; H 3.82; N 11.38. Found: C 52.20; H 3.90; N 11.65.

##### 4-Butyl-1-(3-trifluoromethylbenzoyl)thiosemicarbazide (**3c**)

Yield 67%, m.p. 161.6 °C, ^1^H NMR (DMSO-*d*_6_) δ (ppm): 0.87 (t, 3H, CH_3_, *J* = 7.3 Hz), 1.27–1.32 (m, 2H, CH_2_), 1.43–1.52 (m, 2H, CH_2_), 3.43 (d, 2H, CH_2_, *J* = 6.7 Hz), 7.73–7.78 (m, 1H, ArH), 7.94–7.97 (m, 1H, ArH), 9.15–8.20 (m, 2H, ArH), 8.25 (s, 1H, NH), 9.30 (s, 1H, NH), 10.57 (s, 1H, NH). ^13^C NMR (DMSO-*d*_6_) δ (ppm): 14.27, 19.92, 31.39, 48.89, 123.35, 124.94, 128.79, 129.48, 129.52, 130.10, 131.46, 132.36, 165.07, 177.78. Elemental analysis for C_13_H_16_F_3_N_3_OS. Calculated: C 48.89; H 5.05; N 13.16. Found: C 48.98; H 5.30; N 13.45.

##### 1-(3-Trifluoromethylbenzoyl)-4-(3-methylphenyl)thiosemicarbazide (**3d**)

Yield 58%, m.p. 230.7 °C, ^1^H NMR (DMSO-*d*_6_) δ (ppm): 3.34 (s, 3H, CH_3_), 6.96–6.99 (m, 1H, ArH), 7.18–7.23 (m, 2H, ArH), 7.33–7.36 (m, 1H, ArH), 7.77 (t, 1H, ArH, *J* = 7.8 Hz) 7.97 (d, 1H, ArH, *J* = 7.8 Hz), 8.23 (d, 1H, ArH, *J* = 7.8 Hz), 8.29 (s, 1H, ArH), 9.75–9.80 (m, 2H, 2NH), 10.81 (s, 1H, NH). ^13^C NMR (DMSO-*d*_6_) δ (ppm): 21.39, 123.55, 125.04, 125.35, 126.34, 127.16, 128.33, 128.85, 129.32, 130.11, 132.46, 134.02, 137.77, 139.51, 165.17, 182.16. Elemental analysis for C_16_H_14_F_3_N_3_OS. Calculated: C 54.38; H 3.99; N 11.89. Found: C 54.60; H 4.20; N 11.90.

##### 1-(3-Trifluoromethylbenzoyl)-4-(3-fluorophenyl)thiosemicarbazide (**3e**)

Yield 62%, m.p. 174.4 °C, ^1^H NMR (DMSO-*d*_6_) δ (ppm): 7.62 (t, 1H, ArH, *J* = 8.2 Hz) 7.77–7.82 (m, 1H, ArH) 7.98–8.01 (m, 3H, ArH) 8.25 (d, 1H, ArH, *J* = 7.9 Hz) 8.30 (s, 1H, ArH) 8.43 (s, 1H, ArH) 10.15 (s, 2H, 2NH) 10.93 (s, 1H, NH). ^13^C NMR (DMSO-*d*_6_) δ (ppm): 112.94, 121.85, 121.94, 123.52, 125.01, 125.33, 127.14, 128.95, 129.58, 130.18, 132.44, 133.89, 141.38, 141.45, 156.08, 165.16, 181.49. Elemental analysis for C_15_H_11_F_4_N_3_OS. Calculated: C 50.42; H 3.10; N 11.76. Found: C 50.50; H 3.40; N 11.95.

##### 1-(3-Trifluoromethylbenzoyl)-4-(phenyl)thiosemicarbazide (**3f**)

Yield 69%, m.p. 176.4 °C, ^1^H NMR (DMSO-*d*_6_) δ (ppm): 7.17 (t, 1H, ArH, *J* = 7.2 Hz), 7.33 (t, 2H, ArH, *J* = 7.8 Hz), 7.41 (bs, 2H, ArH), 7.77 (t, 1H, ArH, *J* = 7.8 Hz), 7.97 (d, 1H, ArH, *J* = 7.8 Hz), 8.23 (d, 1H, ArH, *J* = 7.9 Hz), 9.78 (s, 1H, NH), 9.87 (s, 1H, NH), 10.83 (s, 1H, NH). ^13^C NMR (DMSO-*d*_6_) δ (ppm): 123.35, 125.04, 125.35, 126.67, 128.53, 128.86, 129.32, 130.12, 132.44, 134.01, 139.64, 165.17, 181.60. Elemental analysis for C_15_H_12_F_3_N_3_OS. Calculated: C 53.09; H 3.56; N 12.38. Found: C 53.30; H 3.90; N 12.45.

##### 4-(3-Chlorophenyl)-5-(3-trifluoromethylphenyl)-2,4-dihydro-3H-1,2,4-triazole-3-thione (**4**)

Yield 65%, m.p. 241.2 °C, ^1^H NMR (DMSO-*d*_6_) δ (ppm): 7.40 (d, 1H, ArH, *J* = 7.7 Hz), 7.54 (t, 1H, ArH, *J* =8.0 Hz), 7.59–7.61 (m, 2H, ArH), 7.65–7.67 (m, 3H, ArH), 7.83 (d, 1H, ArH, *J* =7.0 Hz), 14.31 (s, 1H, NH). ^13^C NMR (DMSO-*d*_6_) δ (ppm): 123.12, 124.42, 125.44, 127.16, 127.52, 128.26, 129.49, 130.18, 130.43, 131.41, 132.84, 133.80, 136.01, 149.65, 169.20. ^19^F NMR (DMSO-*d*_6_) δ(ppm): −61.66. Elemental analysis for C_15_H_9_ClF_3_N_3_S. Calculated: C 50.64; H 2.55; N 11.81 Found: C 50.85; H 2.90; N 11.90.

### 3.2. Predictions of the Biological Activity

Predictions of the potential biological activity of the newly obtained thiosemicarbazide derivatives were carried out using the PASS (Prediction of Activity Spectra for Substances) software (http://www.ncss.com/software/pass/, accessed on 20 October 2022). [41]. The program enables the prediction of biological activity (including pharmacological effects, mechanisms of action, interactions, and toxicity). The principle of operation is based on the assessment of the similarity of the substance to the structures of biologically active compounds found in the program database (more than 46,000 medicines). The use of the above-mentioned software for the purpose of the above research work was determined by its high accuracy of predicting activity, being on average approx. 95.3%.

### 3.3. Anthelmintic Activity Assay

All compounds in the anthelmintic activity study were prepared in the form of a homogeneous suspension. The anthelmintic activity was tested according to the procedure developed by Bogucka-Kock and Kołodziej [69]. The tested compounds were added to the culture of *Rhabditis* sp. at experimentally determined concentrations. After a 24 h exposure, nematodes were observed in terms of development according to the earlier described procedure [42]. LC_50_ values (lethal concentration) were determined for two active substances. It was defined as the amount of the substance causing the death of 50% of nematodes [43].

### 3.4. Antimicrobial Activity Assay

The microdilution method was used to determine the MIC values of newly synthesized compounds. The antimicrobial activity of the thiosemicarbazides series was tested on ten reference bacterial strains purchased from the American Type Culture Collection (ATCC). Seven Gram-positive reference strains (*S. aureus* ATCC 29213, *S. aureus* ATCC 43300, *S. aureus* ATCC 6538, *S. aureus* ATCC 25923, *S. epidermidis* ATCC 12228, *M. luteus* ATCC 10240, and *B. cereus* ATCC 10876) and three Gram-negative reference bacteria (*K. pneumoniae* ATCC 13883, *E. coli* ATCC 25922, *S. typhimurium* ATCC 14028) were used. These bacterial strains are representatives of microorganisms that can cause the most common opportunistic infections among humans. The bacteria were grown on Mueller–Hinton agar (MHA) plates before examination. To perform the assay, all bacteria were placed in sterile saline (0.85% NaCl) to obtain a suspension of the optical density of the 0.5 McFarland standard (1.50 × 10^8^ colony-forming units (CFUs)/mL). A solution of the tested compounds was prepared in dimethyl sulfoxide (DMSO) at a concentration of 50 mg/mL as a stock solution.

A 96-well microtiter plate and microbroth two-fold dilution method was used to determine the minimum inhibitory concentration (MIC) according to the EUCAST (European Committee on Antimicrobial Susceptibility Testing) reference recommendation [70]. The solutions were then diluted with Mueller–Hinton broth (MHB, Biomaxima, Lublin, Poland) medium to obtain a solution with a basic concentration of 1000 µg/mL. Then, using the two-fold dilution method with MHB, the tested compounds were introduced into the wells of the microplate to provide a final concentration in the range of 0.49 to 1000 µg/mL. Then, 2 μL of bacterial inoculum with a density of 0.5 McFarland standard was added into each well. Each well was filled with a total volume of 100 μL. Then, microtiter plates were incubated for 18 ± 2 h at 35 °C. After incubation, the growth of bacteria was monitored by a Biotek ELx800 (Biokom, Janki, Poland) spectrophotometer at 600 nm (OD_600_). The MIC assays were performed in triplicate.

A negative control with MHB and without tested compounds was used. Clinically used anti-bacterial agents, cefuroxime and vancomycin, were included as positive controls for the purpose of comparison in a concentration range from 0.007 to 15.625 µg/mL.

The in vitro antimicrobial activity of the tested compounds was determined by the MIC, which was defined as the lowest concentration of the compound tested at which no visible growth of the test bacteria was observed. The MBC (the minimum bactericidal concentration) is defined as the lowest concentration of antimicrobial agent resulting in reduction in the viability of the initial bacterial inoculum by ≥99.9% on the plate. Generally, an MBC/MIC ratio less than 4 is considered bactericidal for an antimicrobial, and an MBC/MIC > 8 is indicative of bacteriostatic activity [71,72]. For the MBC test, the Mueller–Hinton agar (MHA, Biomaxima, Poland) plate assay was used. MBC was determined after the broth dilution period of an MIC test has been completed. After the MIC detection, 5 µL aliquots from each well were plated on an MHA plate and grown at 35 °C overnight. The MBC values were determined by observing the first clear area on the agar plate that has no observable bacterial growth.

### 3.5. Anticancer Activity

To conduct biological tests two cell lines were used: the human metastatic colon cancer cell lines SW 620 and V79 which are cells exhibiting fibroblast morphology isolated from the lung of a Chinese hamster. Both lines were obtained from American Type Culture Collection (ATCC, Rockville, MD, USA). The culture used to grow the SW 620 cells was prepared in MEM (ThermoSci, Waltham, MA, USA). The V79 cells were cultured in RPMI (Biowest SAS, Nuaillé, France) with the addition of 20 mM HEPES (Biowest, Nuaillé, France), 10% fetal bovine serum (FBS, Sigma-Aldrich, St. Louis, MO, USA), 100 µg/mL of streptomycin, and 100 U/mL of penicillin (Gibco, Grand Island, NY, USA). The eligible level of cells confluence was 80–90%, and both cell cultures were maintained in a humidified incubator set at 37 °C with 5% CO_2_ until they reached it.

The MTT assay was used to establish the cytotoxic activity of the studied compounds. The mechanism of this method relies on enzymatic conversion of 3-(4,5-dimethylthiazol-2-yl)-2,5-diphentyltetrazolium bromide salt (MTT) into insoluble formazan crystals by mitochondrial dehydrogenases within viable cells.

The process starts with the preparation of 96-well plates by placing cells at a density of 1 × 104 per well and incubating them for 24 h to adhere. Afterwards, the cells were treated with different concentrations of thiosemicarbazide derivatives, from 20 to 100 µM, except the control group which remained not exposed. Next, cells were incubated for 72 h at 37 °C with a 5% CO_2_. Then, a solution of MTT (0.5 mg/mL) was added to each well and plates were incubated for 4 h more in the same conditions.

To dissolve precipitated formazan crystals, a mixture of DMSO and isopropanol (in a 1:1 ratio, *v*/*v*) was used. For the spectrophotometric survey, we used MultiscanGo from Thermo Scientific with the wavelength value set at 570 nm to measure absorbance.

Cell viability was determined as the percentage of MTT reduction in the treated cells compared to the control group (unexposed cells). The relative MTT level, represented as a percentage, was calculated using the formula [A]/[B] × 100, where [A] stands for the absorbance of the treated cell sample and [B] for the absorbance of unexposed to studied compounds sample (control group). Reduced cell viability reveals itself as decreased relative MTT levels. To calculate IC_50_ values (concentration at which 50% of cell viability is inhibited), we used GraphPad Prism 8.0.1 software.

### 3.6. Antioxidant Activity

The antioxidant activity of the tested compounds was measured in the brain homogenate of intact rats bred at the Central Laboratory of Experimental Animals of the Medical University of Warsaw. Brains were collected according to procedure no. 3R. A 10% (*m*/*v*) rat brain solution was obtained by homogenizing freshly prepared brain washed with cold 0.9% NaCl in cold 10 mM TRIS-HCl buffer, with pH = 7.5. The homogenate was centrifuged at 4 °C (2000 rpm) to obtain supernatant 1 [54,60]. Sample preparation was as follows: 100 μM stock solutions of test compounds in DMSO were added to 200 μM of supernatant 1 to a final concentration of 10 μM. The reference sample contained brain homogenate supernatant and DMSO.

#### 3.6.1. Estimation of Lipid Peroxidation

Freshly prepared solutions of 0.1% FeCl_2_ (0.2 mL) and 1 mM ascorbic acid (0.4 mL) [60,73] were added to all samples to initiate the oxidation reaction. Samples were incubated for 1 h at 37 °C. Then, 8.1% sodium dodecyl sulfate (SDS) solution was added to inhibit the oxidation reaction. Acetate buffer (pH = 3.4) and 0.8% thiobarbituric acid (TBA) solution (pH = 6) were added to each sample to determine the concentration of malondialdehyde (MDA). The samples were incubated for 1 h at 100 °C, then cooled on ice and centrifuged (10 min, 4000 rpm). The absorbance was measured at a wavelength of 532 nm against a blank sample (acetate buffer with TBA solution) [60,74,75,76,77,78].

Calculation of malondialdehyde (MDA) concentration [60] was performed as follows:

$$MDA\;concetration=∆\;A_{532\;nm}\times 1.56\times 10^{-6}\;[\frac{\frac{nmol\;MDA}{h}}{g\;tissue}]$$

#### 3.6.2. Statistical Analyses

Statistical analyses were performed using GraphPad Prism 9 software (GraphPad Software, San Diego, CA, USA). The results are expressed as mean ± SD from at least three independent experiments. The statistical significance of differences between means was established by ANOVA with Dunnett’s multiple comparison pos hoc test. *p* values below 0.05 were considered statistically significant.

### 3.7. The Lipinski Rules

The Lipinski rule, also known as the “Rule of Five”, is a guideline used in drug discovery to assess the likelihood of a drug candidate’s success as an orally administered medication [79,80,81]. The rule states that a compound is more likely to be orally active if it has:

1. No more than five hydrogen bond donors (i.e., NH or OH groups);
2. No more than ten hydrogen bond acceptors (i.e., N or O atoms);
3. A molecular weight of less than 500 Daltons;
4. A partition coefficient (log P) of less than 5;
5. Molar refractivity ranging from 40 to 130.

The rule does not state that a medicine that is active when taken orally will be tiny and only mildly lipophilic. Rather, it suggests that a compound is more likely to be orally active if it has moderate lipophilicity, as indicated by a log *p* value between −0.4 and 5.0. Compounds that are too hydrophilic or too lipophilic may have poor oral absorption, distribution, metabolism, and excretion (ADME) properties, which can limit their effectiveness as oral medications. However, lipophilicity is just one of several factors that influence a drug’s oral bioavailability, and other physicochemical and pharmacological properties must also be considered in drug discovery and development. Molinspiration (http://www.molinspiration.com, accessed on 20 March 2023) is used to estimate the drug-likeness qualities of the compounds by verifying Lipinski’s rule of five.

## 4. Conclusions

The results of the research showed that the newly obtained compounds with thiosemicarbazide skeleton had anthelmintic and antibacterial potential. Moreover, the compounds did not show significant cytotoxic activity towards the tested cancer line and normal cell line. The most active was thiosemicarbazide with a 3-chlorophenyl substituent (**3a**). In fact, the efficacy of these compounds was found to be higher than that of the therapeutics currently used in the treatment of parasitic diseases. Additionally, all compounds showed antioxidant activity, but the most active was a molecule with a fluorine atom in the phenyl ring (**3e**). Analysis of the assumptions of the rule of five indicates that all obtained compounds should be characterized by good bioavailability. Our research shows that the newly synthesized compounds may constitute a promising group of compounds for further structural modifications.

## Figures and Tables

**Table 1:** The probability of the predicted biological activity of the synthesized thiosemicarbazides.

Compound	Antituberculosis	Antibacterial	Antiparasitic
**3a**	0.551	0.381	0.441
**3b**	0.513	0.337	0.341
**3c**	0.479	0.440	0.390
**3d**	0.555	0.389	0.328
**3e**	0.541	0.363	0.269
**3f**	0.594	0.414	0.335

**Table 2:** The antimicrobial activity results of the newly synthesized compounds against reference strains of Gram-positive bacteria expressed as the values of the minimal inhibitory concentration (MIC; µg/mL) and the minimal bactericidal concentration (MBC; µg/mL).

Compounds	*Staphylococcus aureus* ATCC 25923		*Staphylococcus aureus* ATCC 29213		*Staphylococcus aureus* ATCC 6538		*Staphylococcus aureus* ATCC 43300		*Staphylococcus epidermidis* ATCC 12228		*Micrococcus luteus* ATCC 10240		*Bacillus cereus* ATCC 10876	
	MIC	MBC	MIC	MBC	MIC	MBC	MIC	MBC	MIC	MBC	MIC	MBC	MIC	MBC
**3a**	1.95	1000	1.95	1000	1.95	1000	3.9	1000	1.95	15.63	1.95	15.63	1.95	15.63
**3b**	62.5	nd *	>1000	nd *	>1000	nd *	->1000	nd *	>1000	nd *	500	nd *	>1000	nd *
**3c**	250	nd *	>1000	nd *	->1000	nd *	->1000	nd *	1000	nd *	1000	nd *	>1000	nd *
**3d**	1000	nd *	>1000	nd *	->1000	nd *	->1000	nd *	1000	nd *	1000	nd *	>1000	nd *
**3e**	31.25	>1000	15.63	>1000	15.63	>1000	31.25	>1000	31.25	>1000	15.63	>1000	7.81	>1000
**3f**	62.5	nd *	>1000	nd *	>1000	nd *	>1000	nd *	1000	nd *	1000	nd *	>1000	nd *
**4**	1000	nd *	>1000	nd *	>1000	nd *	>1000	nd *	500	nd *	1000	nd *	>1000	nd *
^1^ Cef	0.49		nt		0.98		nt		0.25		0.25		31.25	
^2^ Van	2	2					2	2						

^1^ Cef—cefuroxime, ^2^ Van—vancomycin, nd *—not determined, nt—not tested.

**Table 3:** Cytotoxic activity of studied compounds estimated by MTT assay ^a^.

	Cancer Cells		Normal Cells
	SW 620 ^d^		V 79 ^e^
Compound	IC_50_ ^b^	SI ^c^	IC_50_ ^b^
**3a**	69.02 ± 12.63	0.59	40.49 ± 3.83
**3b**	>100	1	>100
**3c**	>100	1	>100
**3d**	>100	0.69	69.09 ± 5.45
**3e**	89.87 ± 14.61	1.06	>100
**3f**	>100	1	>100
**4**	>100	1	>100
Ref ^f^	1.83 ± 0.10	0.16	0.46 ± 0.03

^a^ The MTT assay is a colorimetric assay for measuring cell metabolic activity. It is based on the ability of nicotinamide adenine dinucleotide phosphate (NADPH)-dependent cellular oxidoreductase enzymes to reduce the tetrazolium dye MTT to its insoluble formazan, which has a purple color. Data are expressed as mean SD. ^b^ IC_50_ (µM)—the concentration of the compound that corresponds to a 50% growth inhibition of cell line (as compared to the control) after the cells were cultured for 72 h with the individual compound. ^c^ The SI (Selectivity Index) was calculated using the following formula: SI = IC_50_ for normal cell line/IC_50_ cancer cell line. ^d^ Human metastatic colon cancer (SW620). ^e^ Lung cells isolated from a Chinese hamster. ^f^ The selected reference compound commonly used in cancer treatment (Doxorubicin).

**Table 4:** Antioxidant potential of tested compounds estimated by malondialdehyde (MDA) concentration in rat brain homogenate.

Compound	MDA [mM × 10^−3^]	Antioxidative Potential Value [%]
**3a**	1.395 ± 0.232 *	48.3
**3b**	1.703 ± 0.177 **	36.9
**3c**	1.773 ± 0.077 ***	34.3
**3d**	1.320 ± 0.143 **	51.1
**3e**	1.274 ± 0.121 **	52.8
**3f**	1.450 ± 0.006 ***	46.3
**4**	2.022 ± 0.121 **	25.1
Control Sample	2.699 ± 0.088	0.0

Data represent the mean ± SD of 3 independent samples. Control sample: oxidation reaction was induced by 0.1% FeCl_2_ and 1 mM ascorbic acid. *** *p* ≤ 0.001, ** *p* ≤ 0.01, * *p* ≤ 0.05, difference as compared to the control sample. The antioxidant potential was determined based on the analysis of MDA concentrations between rat brain homogenates containing the tested compounds and the control sample.

**Table 5:** Lipinski’s parameters for compounds **3a**–**3f**.

Compound	Lipinski’s Parameters					
	TPSA	Hydrogen Bond Acceptor	Hydrogen Bond Donor	log *p*	Mass	Rotatable Bonds (No’s)
**3a**	53.15	4	3	3.67	373.79	6
**3b**	62.39	5	3	3.05	369.37	7
**3c**	53.15	4	3	3.39	319.35	8
**3d**	53.15	4	3	3.44	353.37	6
**3e**	53.15	4	3	3.15	357.33	6
**3f**	53.15	4	3	3.01	339.34	6

## Data Availability

The details of the data supporting the report results in this research were included in the paper and Supplementary Materials.

## Supplementary Materials

The following supporting information can be downloaded at: https://www.mdpi.com/article/10.3390/molecules29071529/s1, Figures S1–S7: ^1^H NMR spectra, Figures S8–S14 ^13^C NMR spectra, Figures S15 and S16 ^19^F NMR spectra. Figure S1. The 1H NMR of compound **3a.** Figure S2. The 1H NMR of compound **3b**. Figure S3. The 1H NMR of compound **3c**. Figure S4. The 1H NMR of compound **3d**. Figure S5. The 1H NMR of compound **3e**. Figure S6. The 1H NMR of compound **3f**. Figure S7. The 1H NMR of compound **4**. Figure S8. The 13C NMR of compound **3a**. Figure S9. The 13C NMR of compound **3b**. Figure S10. The 13C NMR of compound **3c**. Figure S11. The 13C NMR of compound **3d**. Figure S12. The 13C NMR of compound **3e**. Figure S13. The 13C NMR of compound **3f**. Figure S14. The 13C NMR of compound **4**. Figure S15. The 19F NMR of compound **3a**. Figure S16. The 19F NMR of compound **4**.
