# Supplementary material for: New Thiosemicarbazide Derivatives with Multidirectional Biological Action
Source: Molecules. 2024 Mar 29;29(7):1529. doi: 10.3390/molecules29071529 (PMC11013662; doi:10.3390/molecules29071529)
Supplement: Supplementary file 1 [file molecules-29-01529-s001.zip › molecules-2926381-supplementary.pdf]

Supplementary material

Patryk Lasek[1], Urszula Kosikowska[2], Przemysław Kołodziej[3], Grażyna Kubiak-Tomaszewska[4], Natalia Krzyżanowska[3], Tomasz Szostek[4], Marta Struga[4], Marcin Feldo[5], Anna Bogucka-Kocka[3], Monika Wujec[6*],

[1]Doctoral School, Medical University of Lublin, Chodzki 7, 20-093 Lublin, Poland; lasekpatryk2@gmail.com

[2]Department of Pharmaceutical Microbiology, Faculty of Pharmacy, Medical University, 20-093 Lublin, Poland; urszula.kosikowska@umlub.pl

[3]Department of Biology and Genetics, Faculty of Pharmacy, Medical University of Lublin, 4A Chodzki Street, 20-093 Lublin, Poland; przemyslawkolodziej@umlub.pl, natalia.krzyzanowska97@gmail.com, anna.bogucka-kocka@umlub.pl

[4]Department of Biochemistry, Medical University of Warsaw, 02-097 Warszawa, Poland; grazyna.kubiak-tomaszewska@wum.edu.pl, tomasz.szostek@wum.edu.pl; marta.struga@wum.edu.pl

[5]Department of Vascular Surgery, Medical University of Lublin, Staszica 11 St., 20-081 Lublin, Poland, marcin.feldo@umlub.pl

[6]Department of Organic Chemistry, Faculty of Pharmacy, Medical University of Lublin, 4A Chodzki Street, 20-093 Lublin, Poland

*Correspondence: monika.wujec@umlub.pl (M.W.)

Content

1. [1]NMR spectra of the compounds……………………………………2

2. ^{13}C NMR spectra for the compounds………………………………..9

3. ^{19}F NMR spectra for compounds **3a** and **4**…………………………15

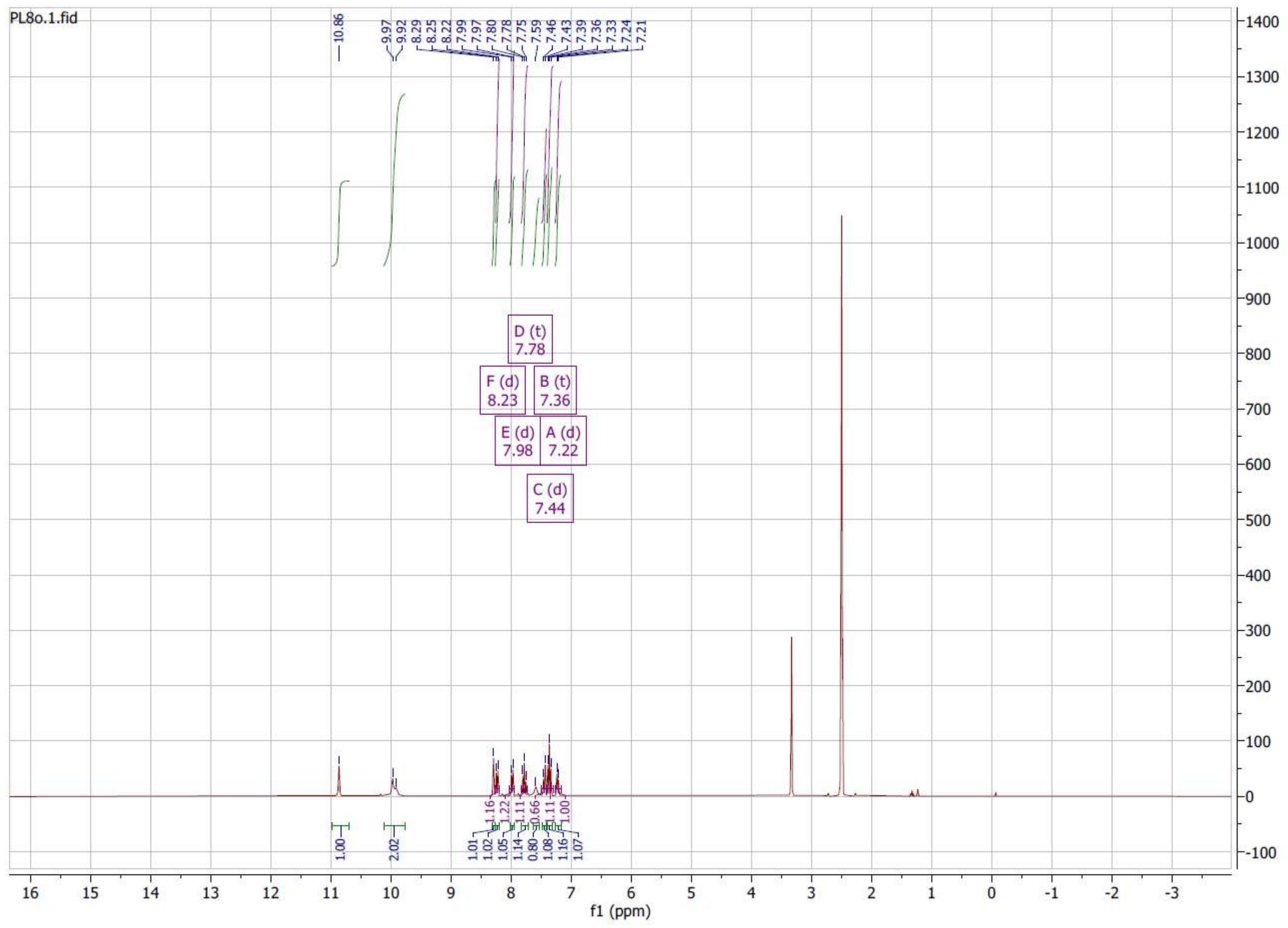

Figure S1. The ^{1}H NMR of compound **3a**.

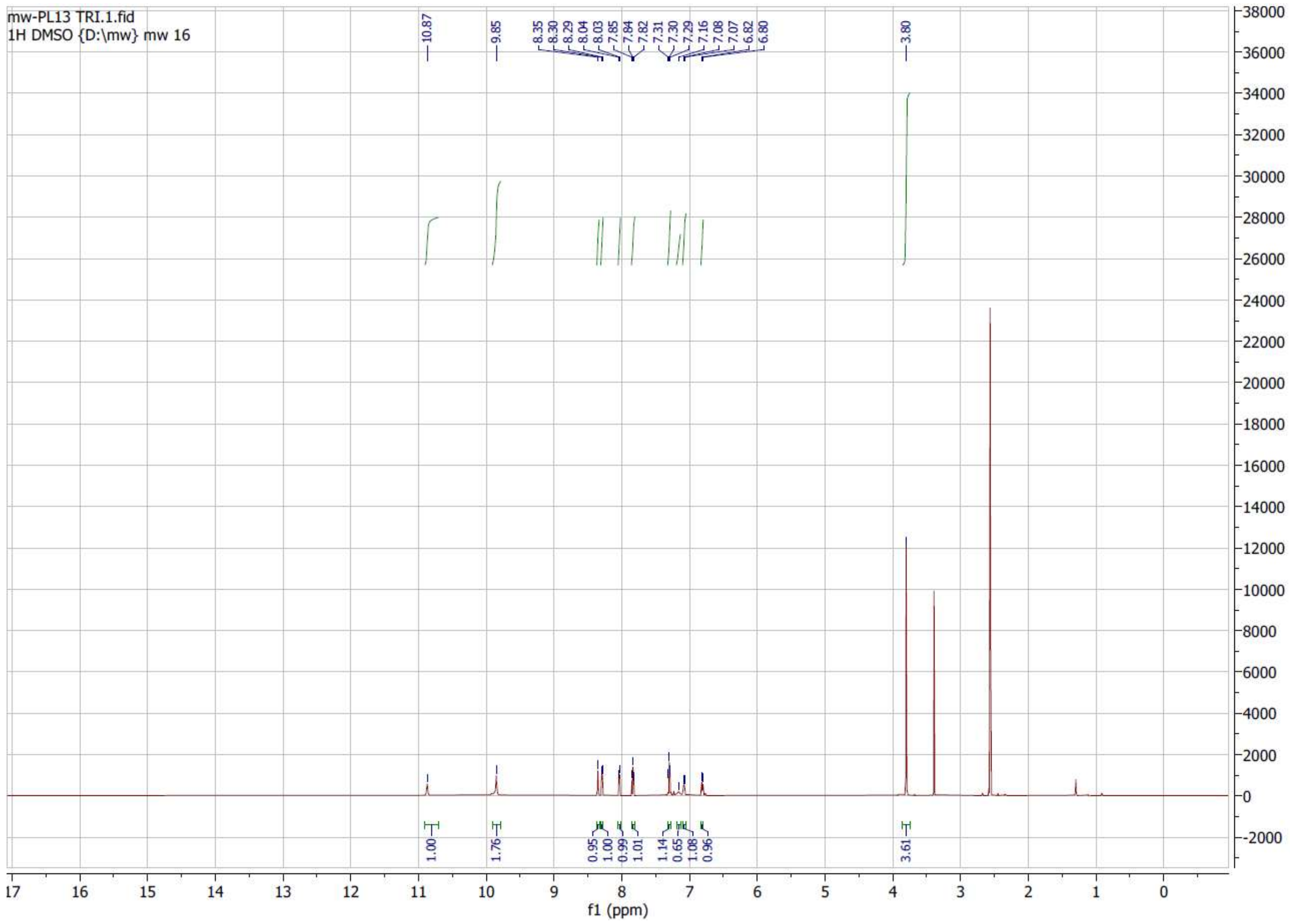

Figure S2. The ^{1}H NMR of compound **3b**.

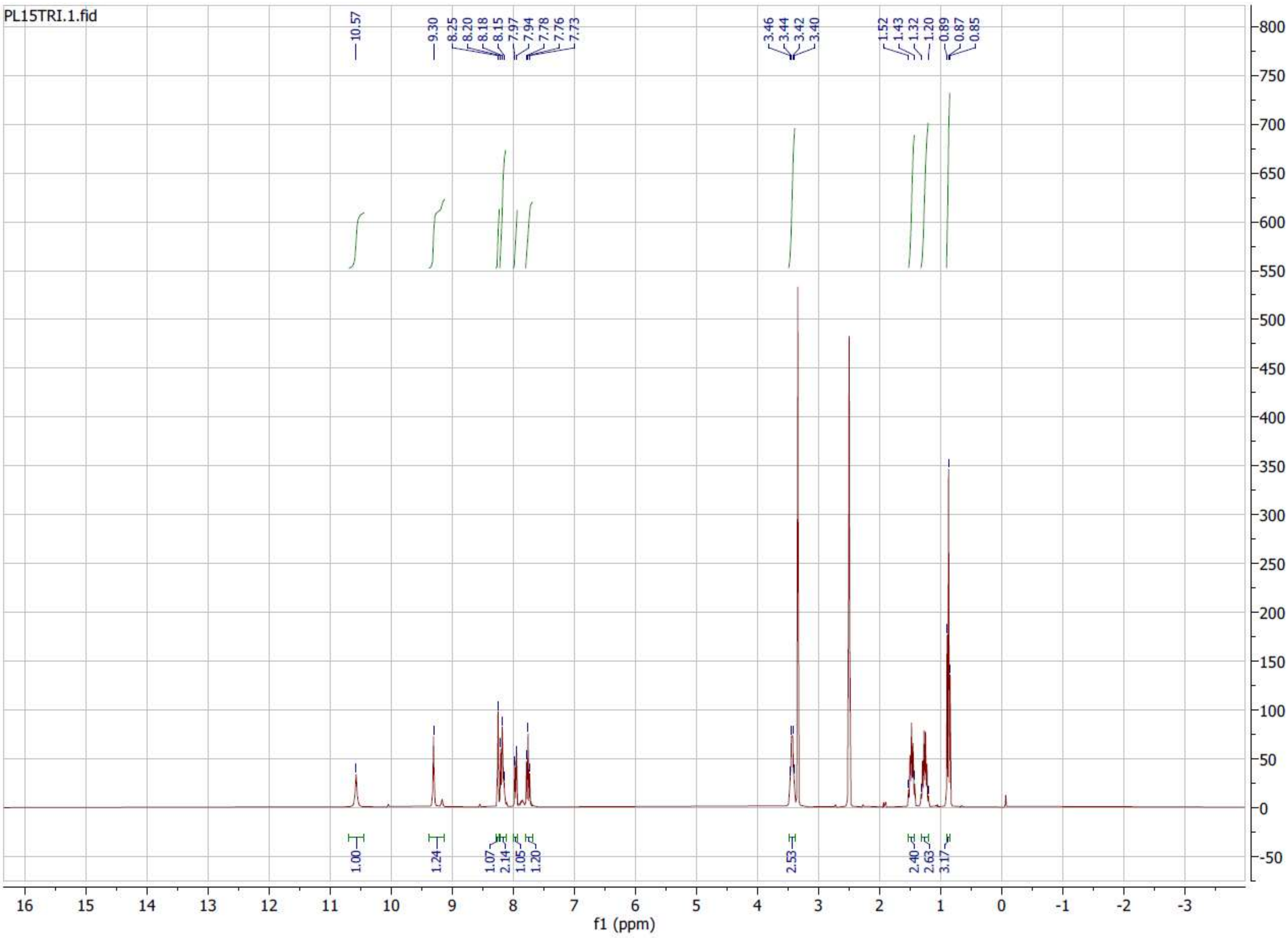

Figure S3. The ^{1}H NMR of compound **3c**.

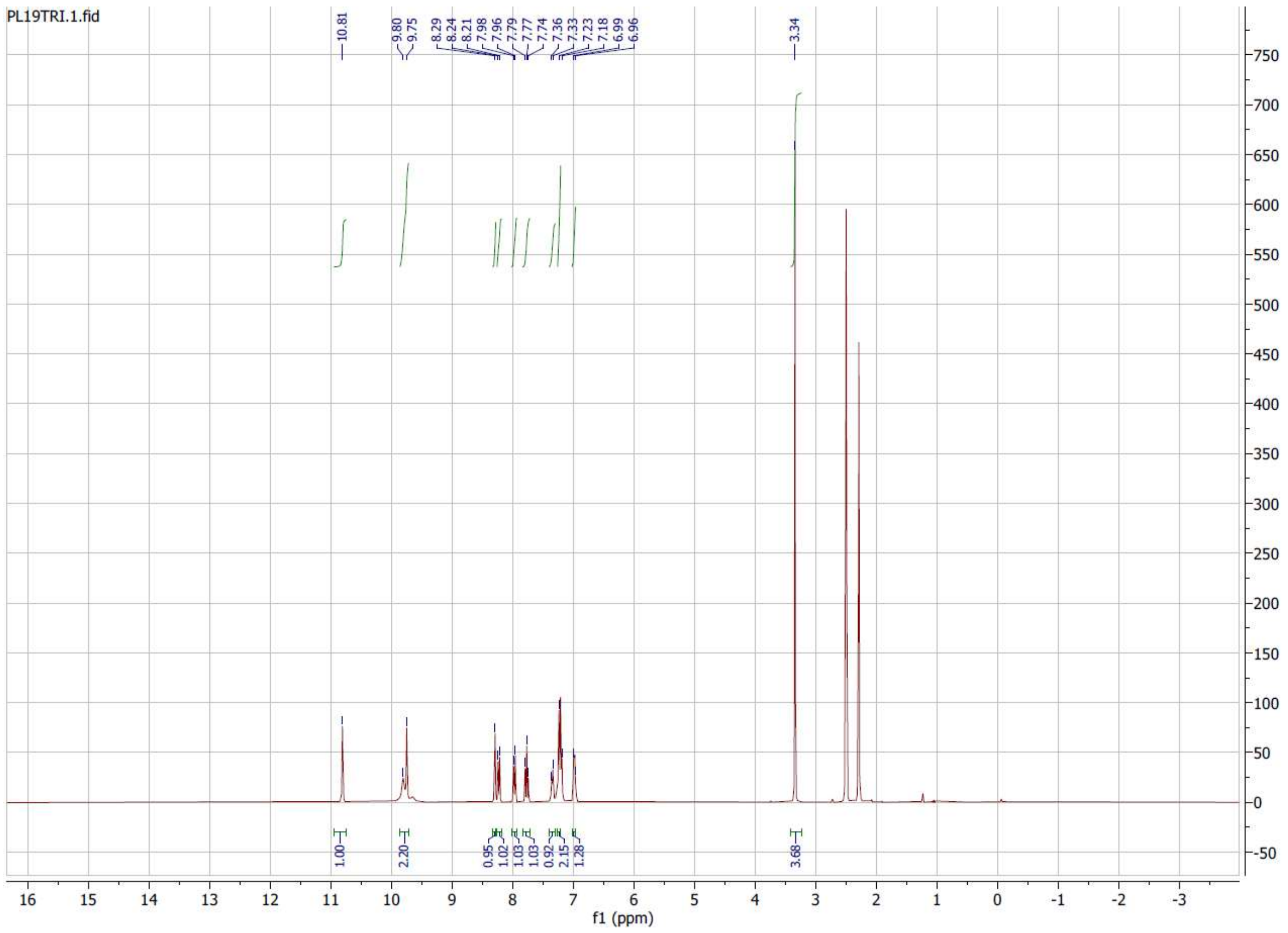

Figure S4. The ^{1}H NMR of compound **3d**.

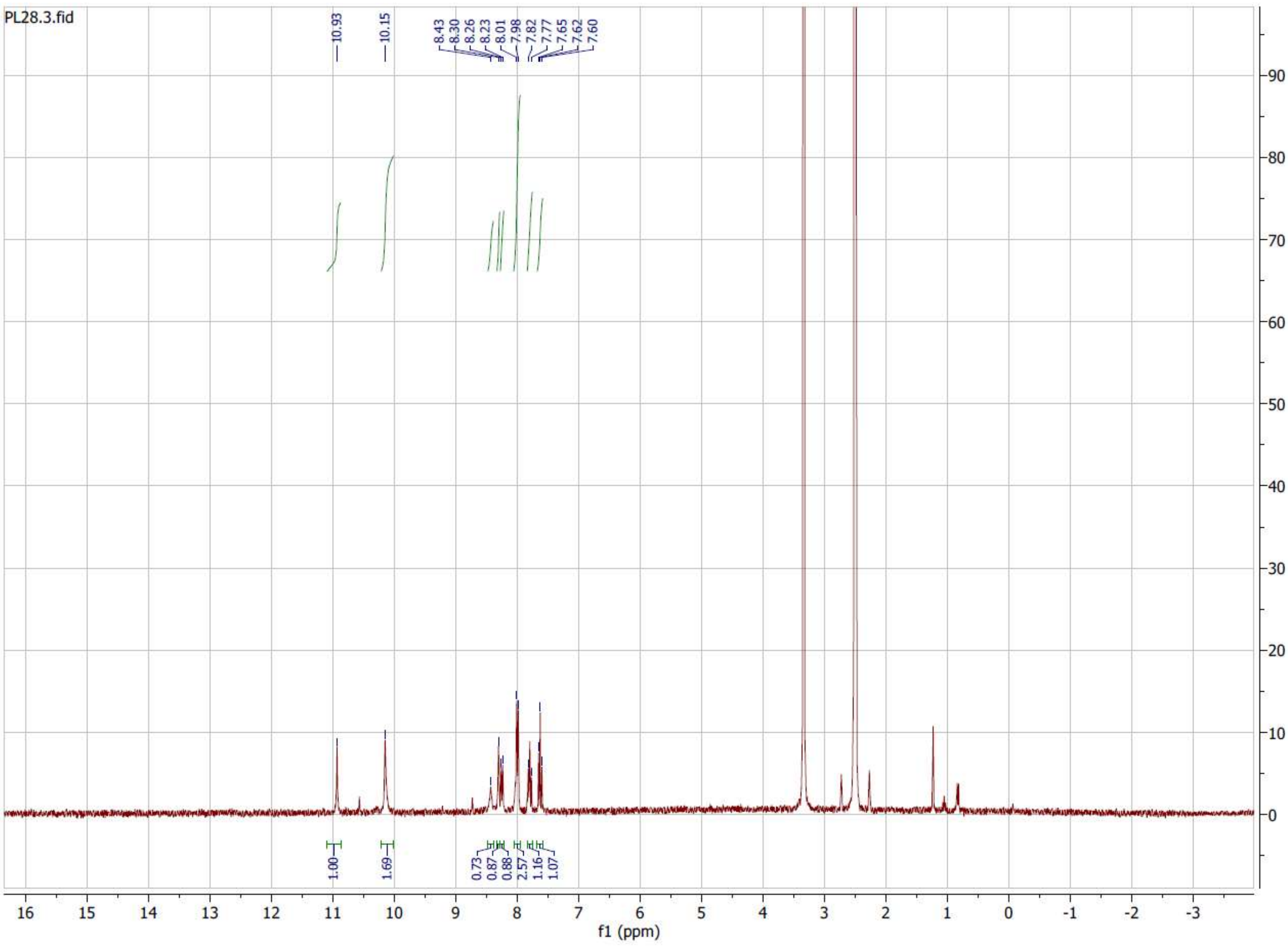

Figure S5. The ^{1}H NMR of compound **3e**.

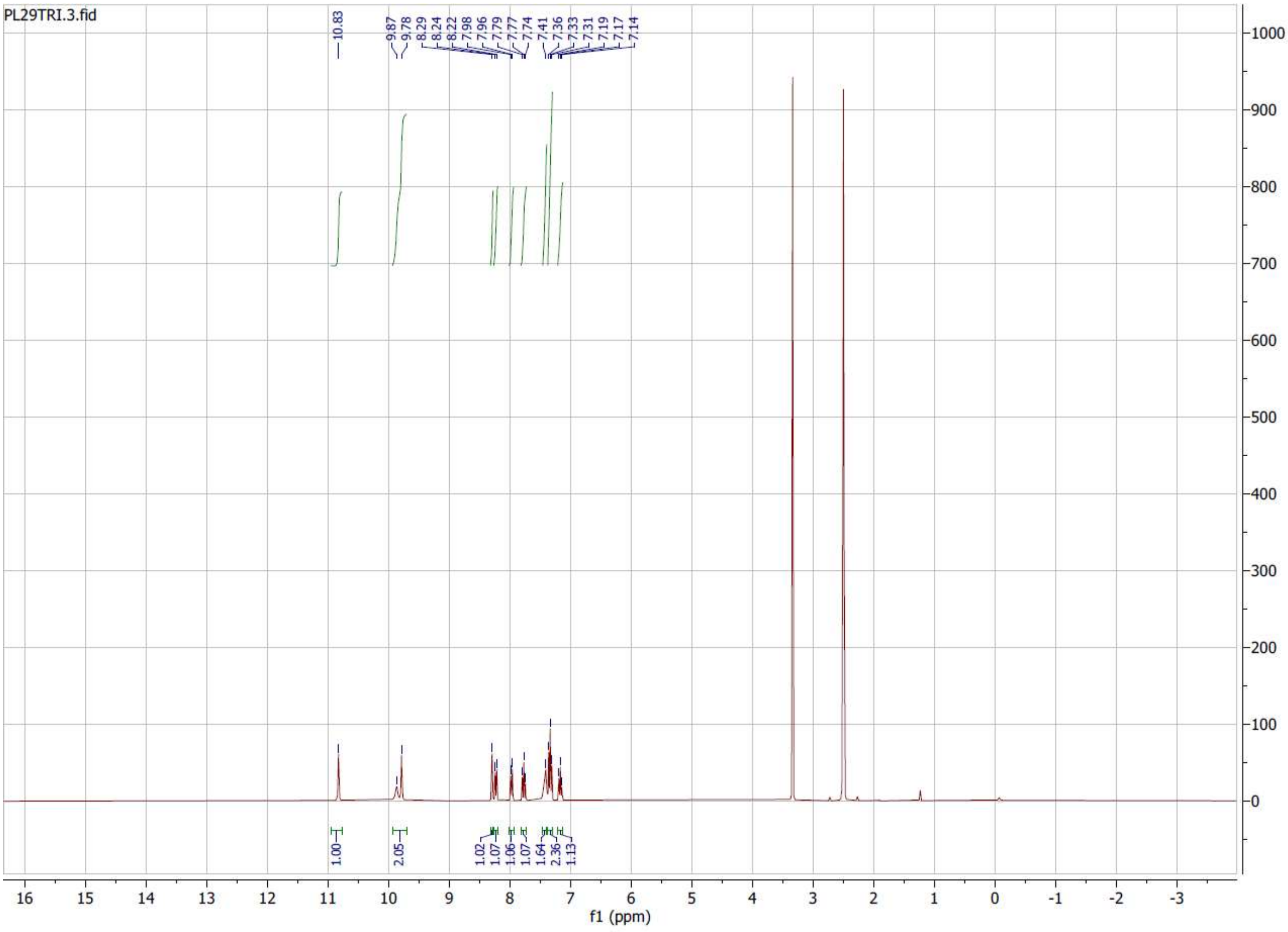

Figure S6. The ^{1}H NMR of compound **3f**.

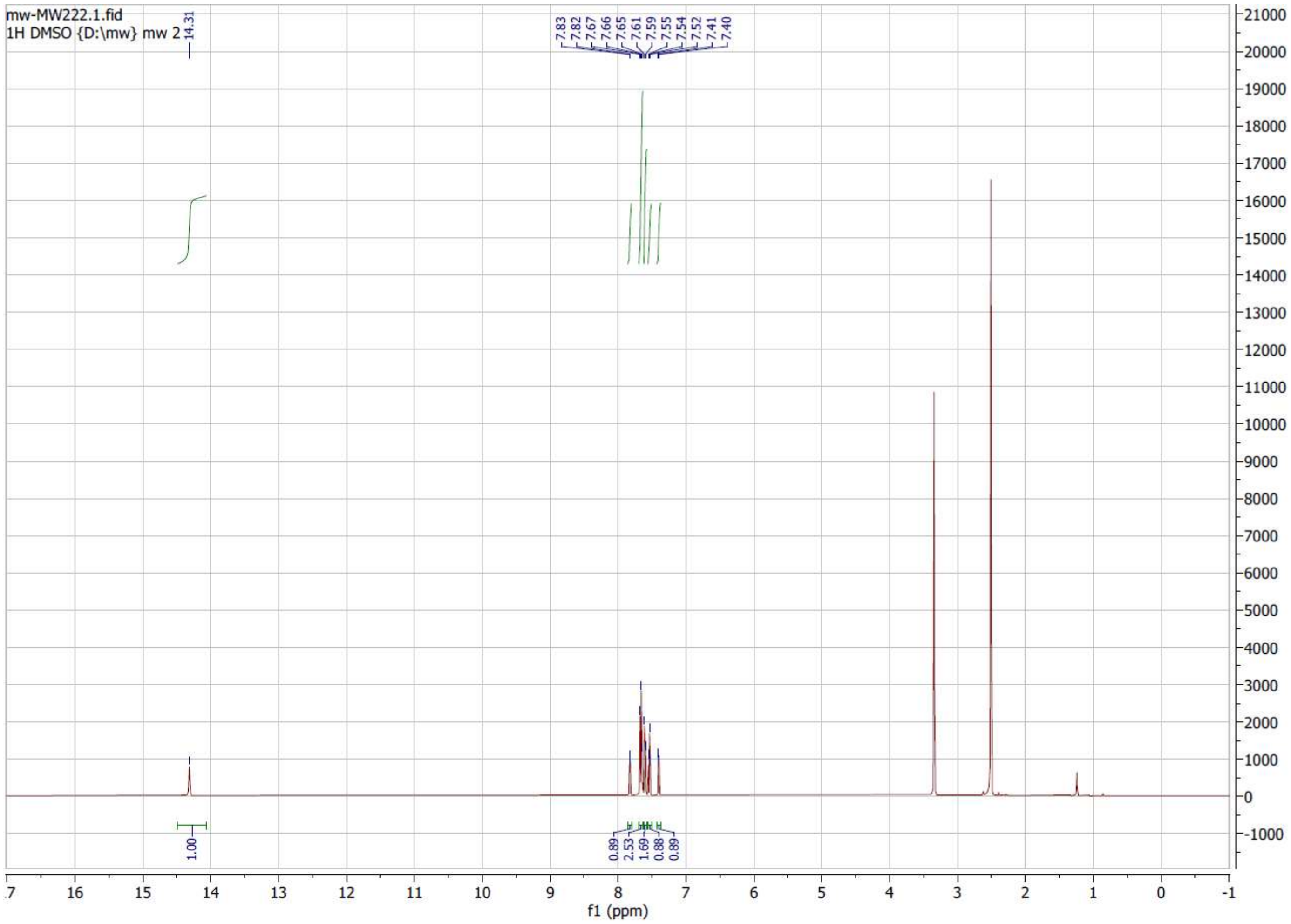

Figure S7. The ^{1}H NMR of compound **4**.

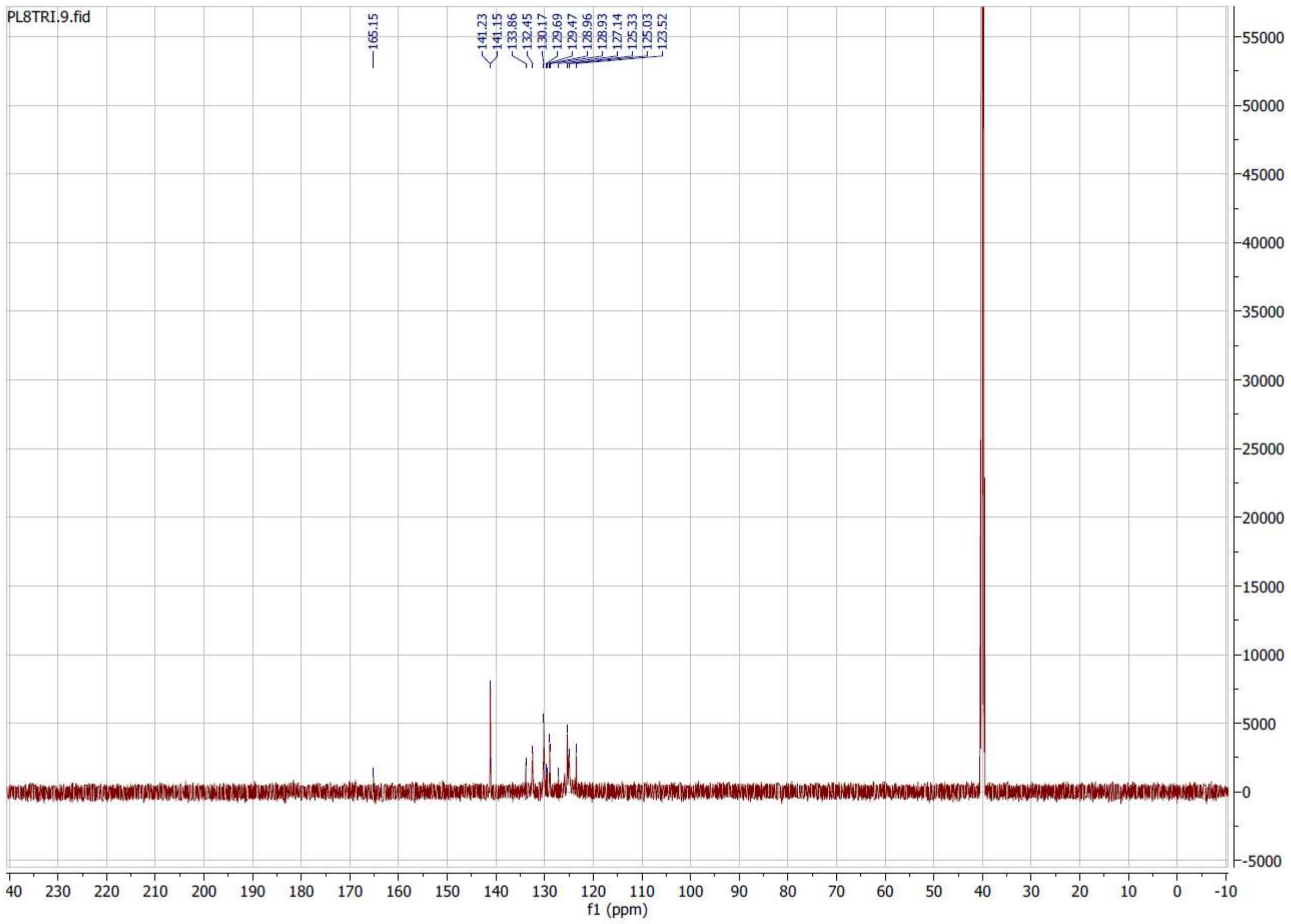

Figure S8. The ^{13}C NMR of compound **3a**.

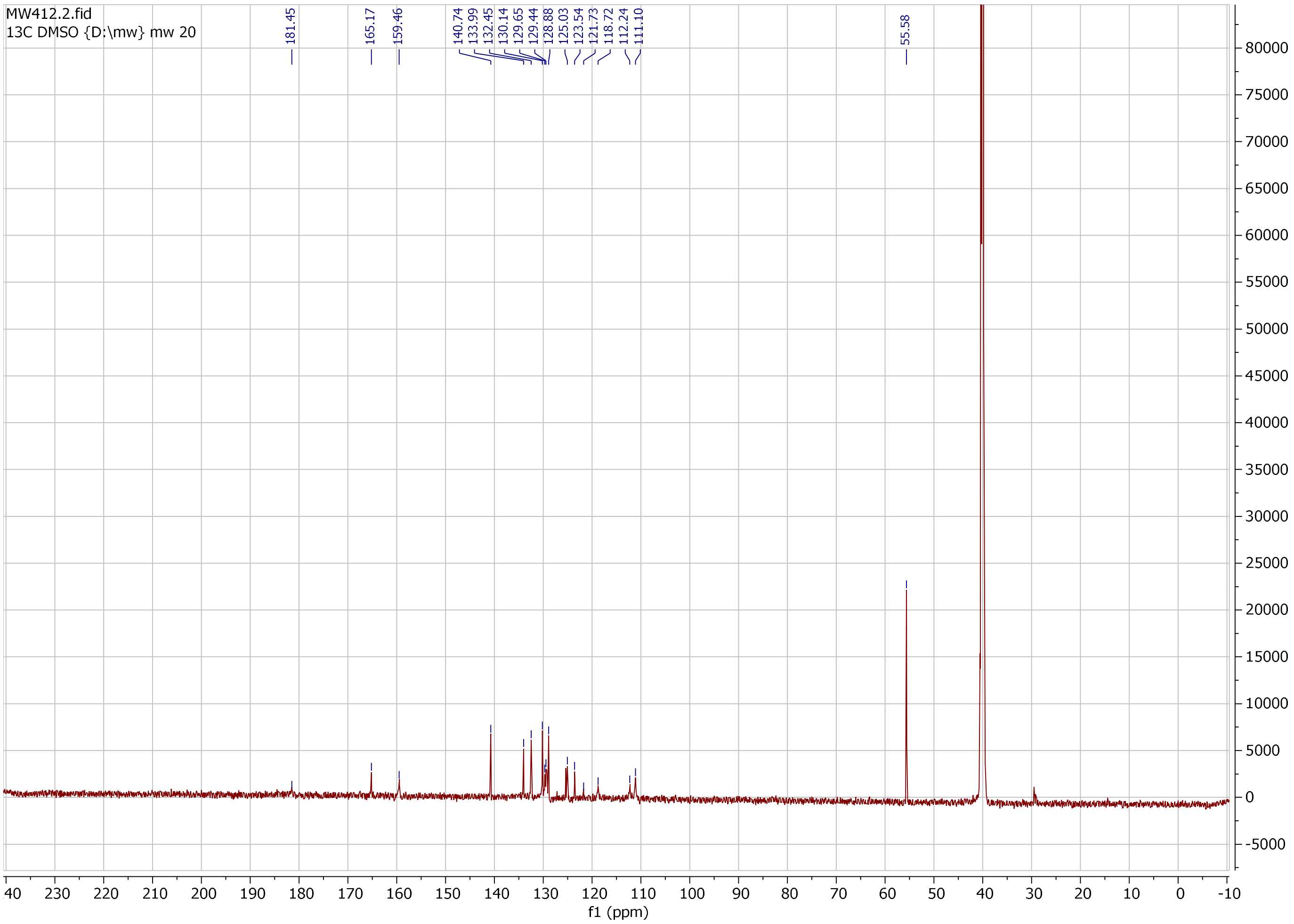

Figure S9. The ^{13}C NMR of compound **3b**.

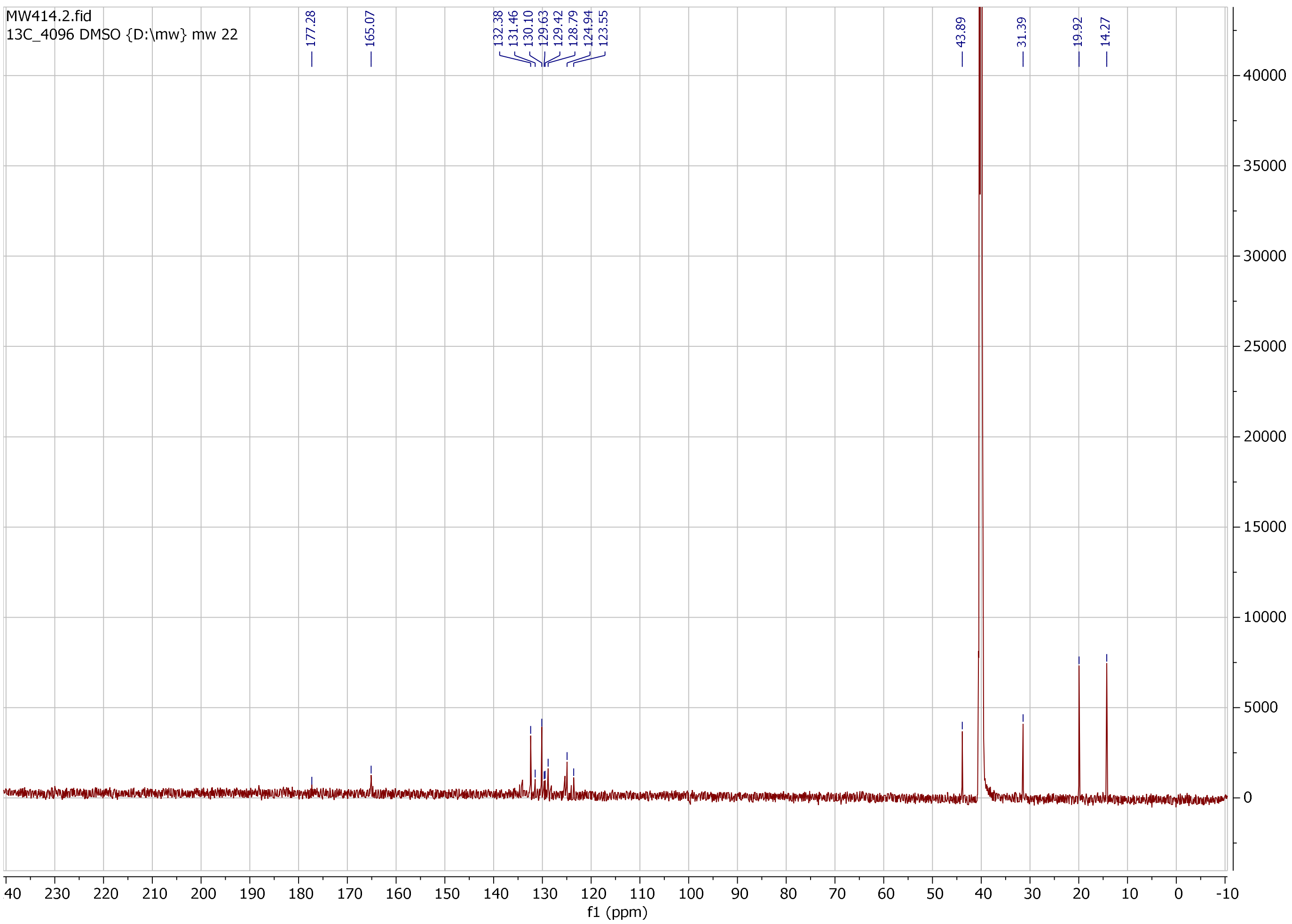

Figure S10. The ^{13}C NMR of compound **3c**.

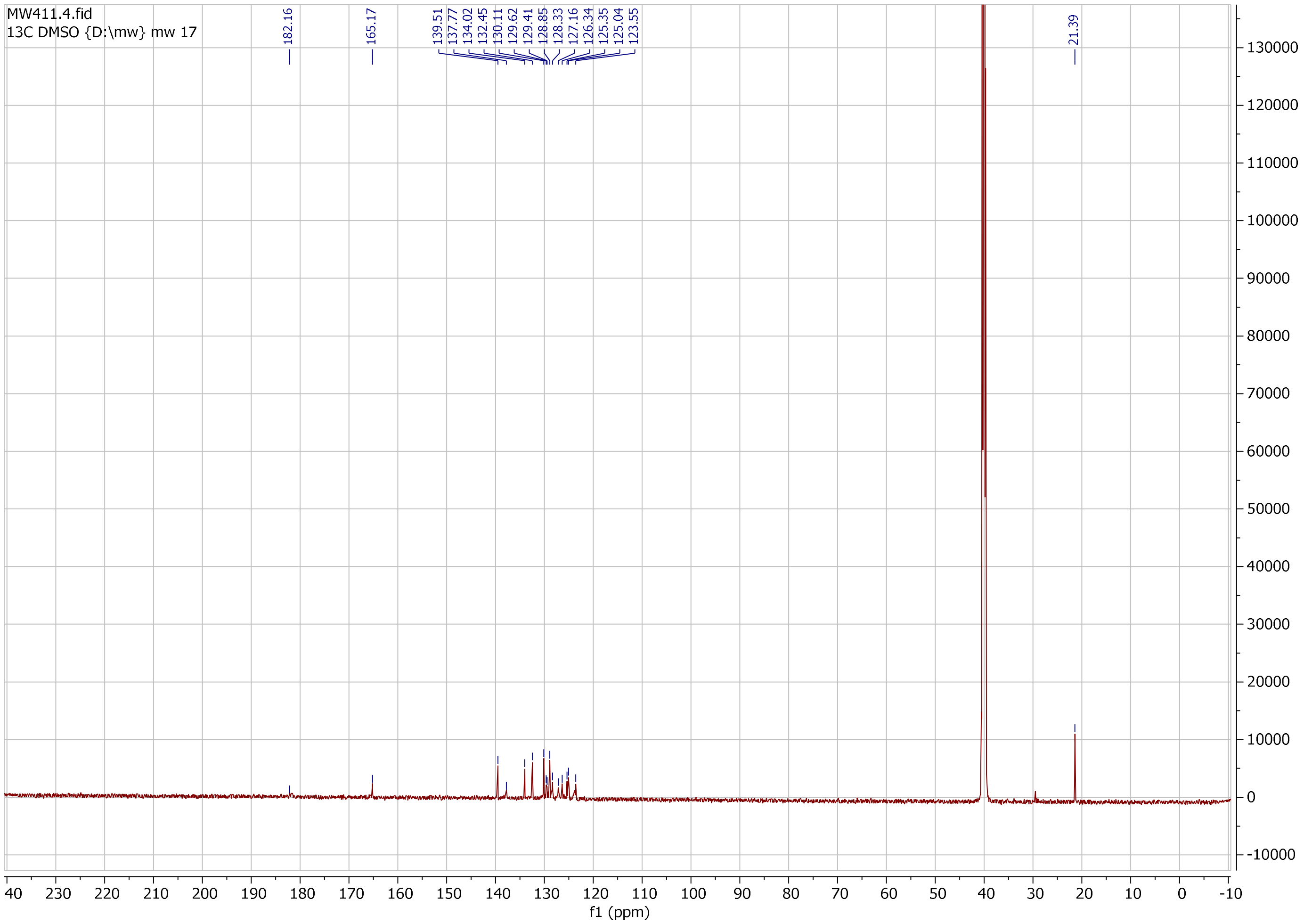

Figure S11. The ^{13}C NMR of compound **3d**.

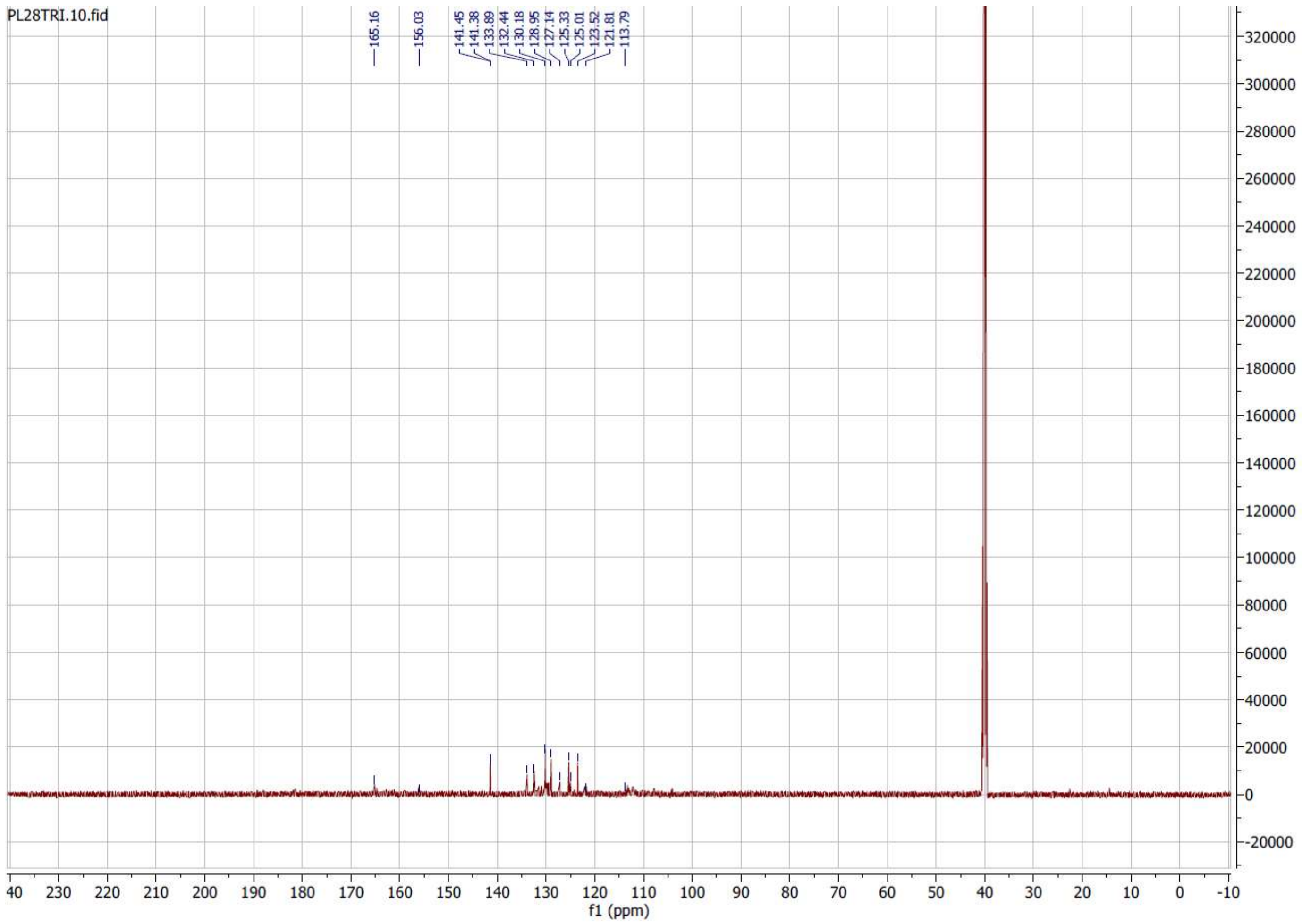

Figure S12. The ^{13}C NMR of compound **3e**.

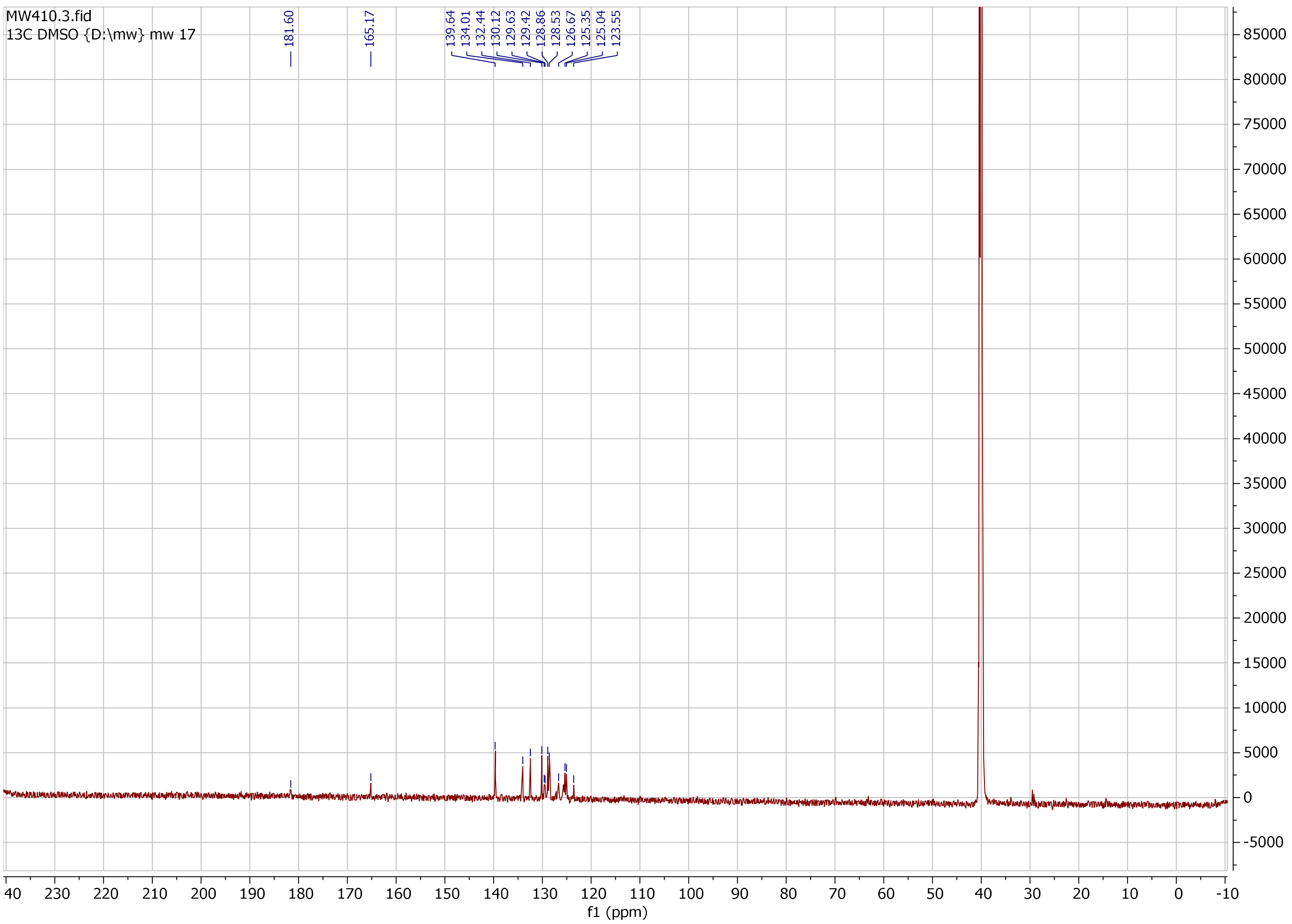

Figure S13. The ^{13}C NMR of compound **3f**.

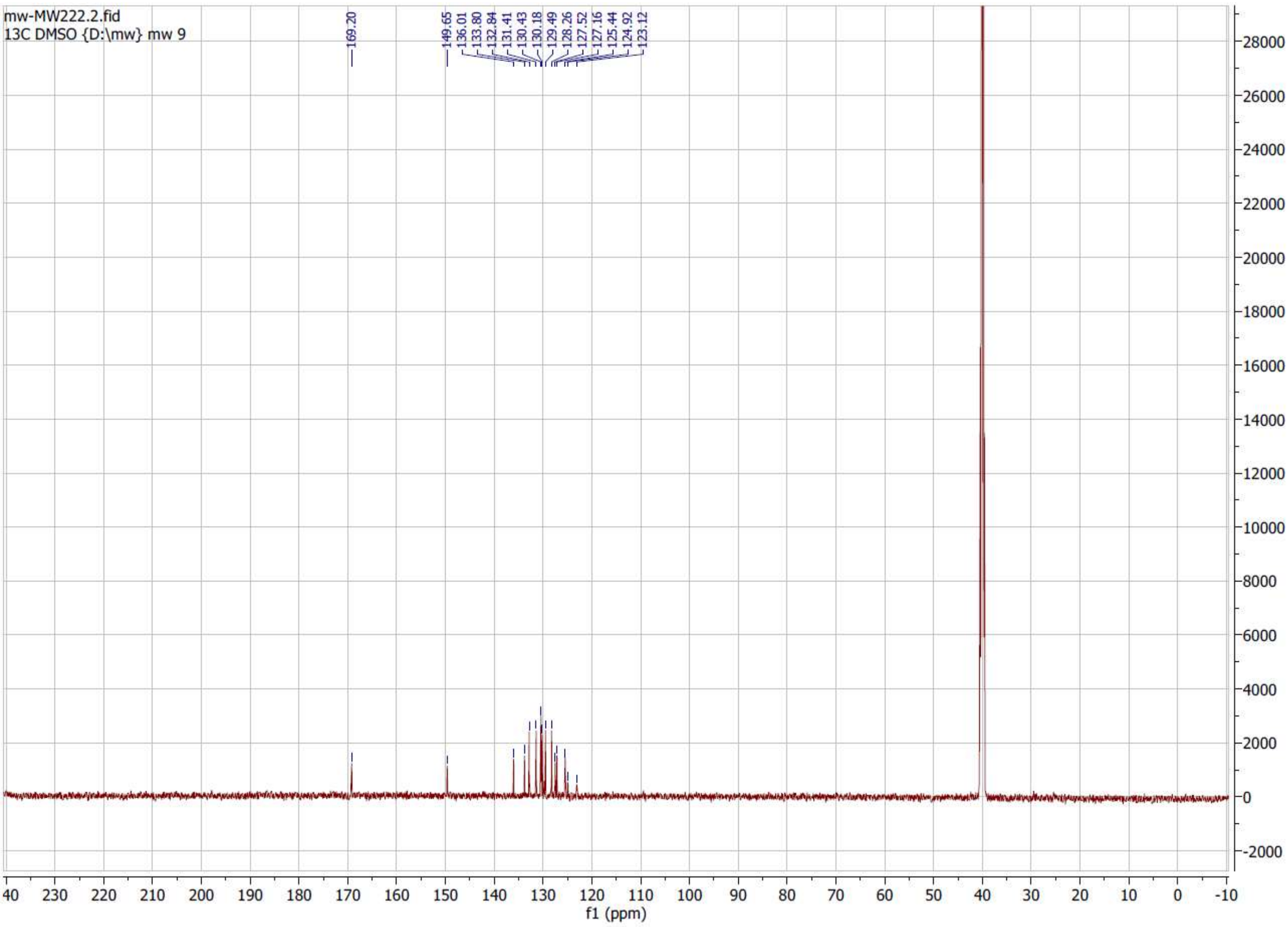

Figure S14. The ^{13}C NMR of compound **4**.

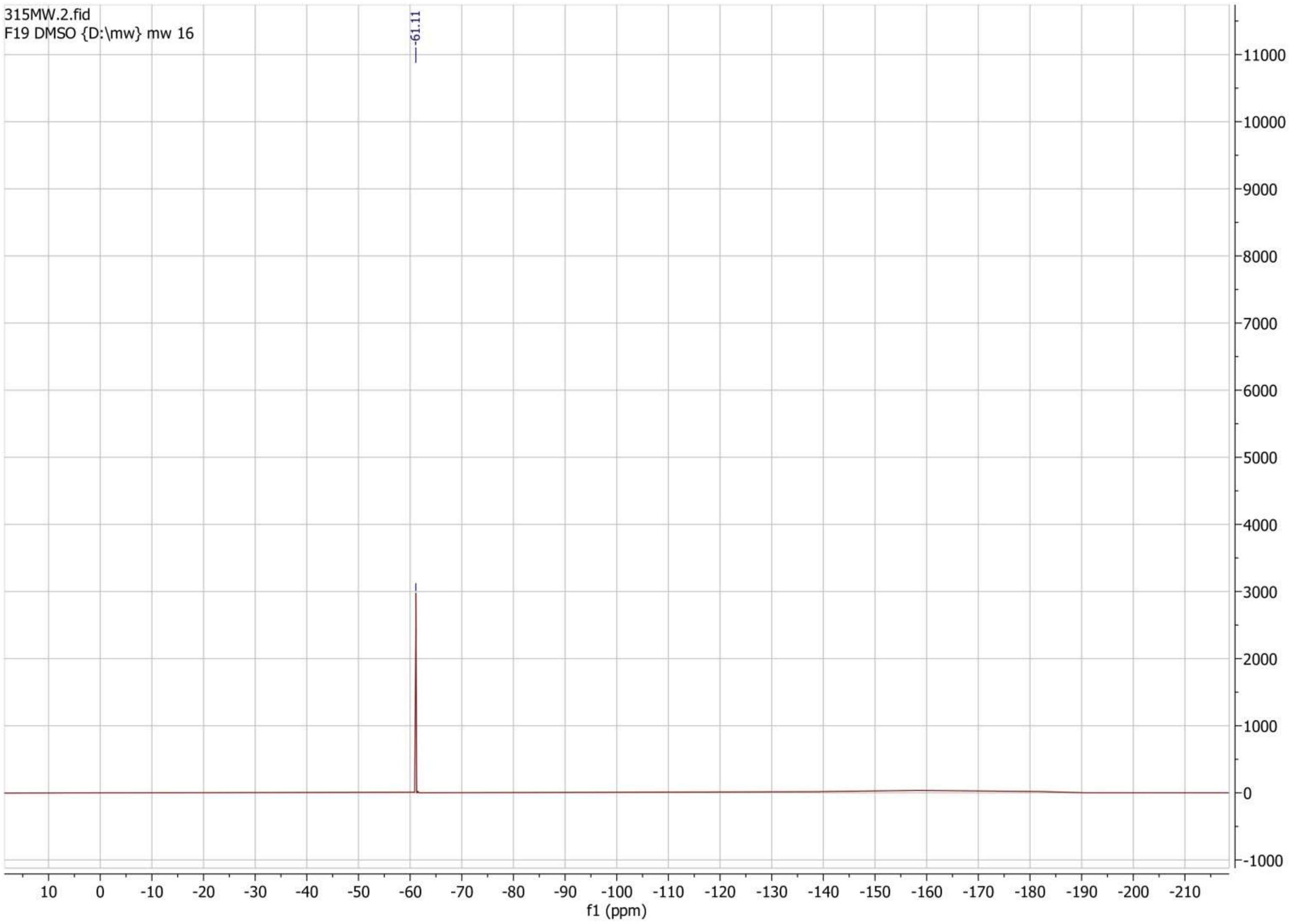

Figure S15. The ^{19}F NMR of compound **3a**.

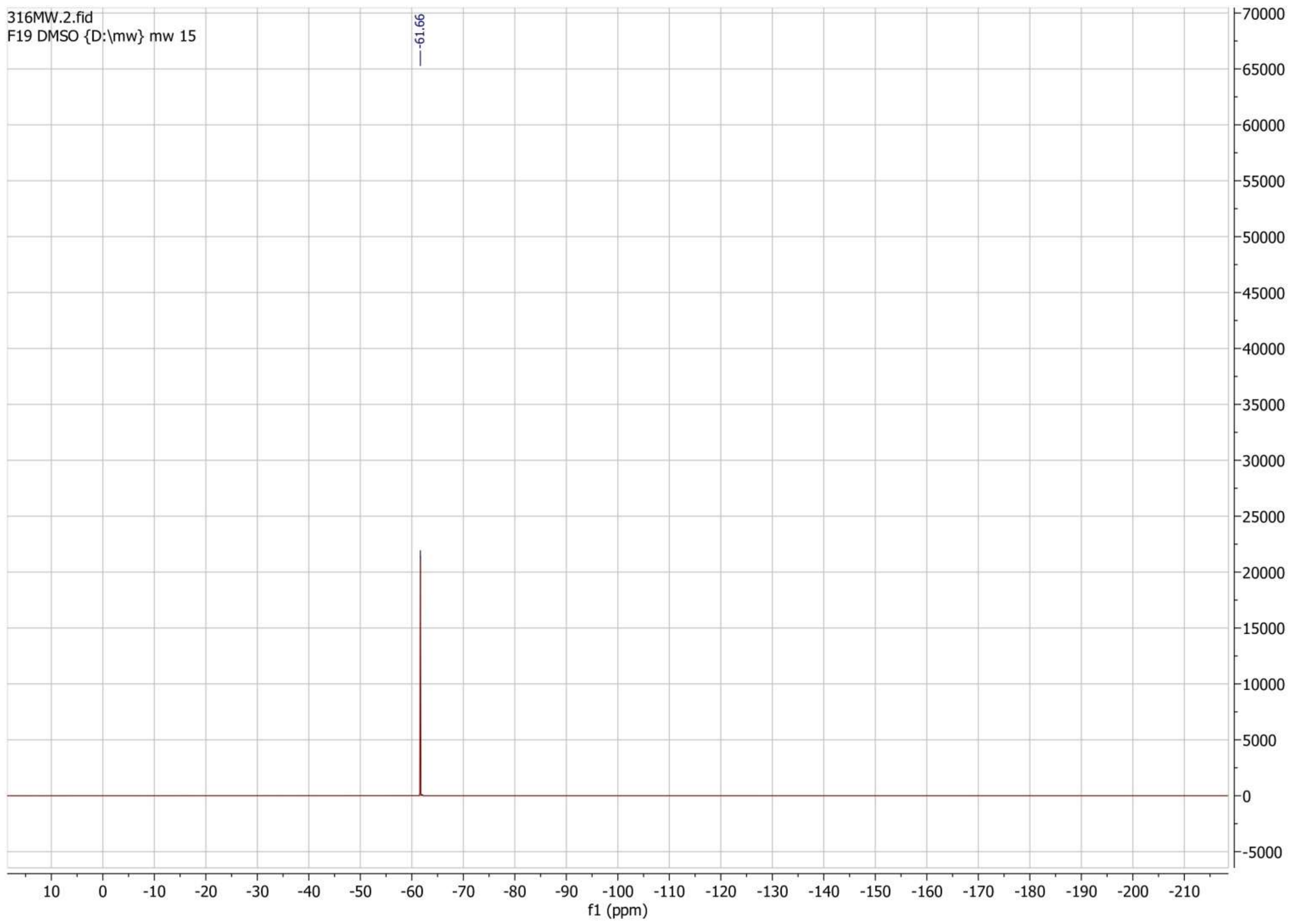

Figure S16. The ^{19}F NMR of compound **4**.
